# Desymmetrisation of *meso*-diones promoted by a highly recyclable polymer-supported chiral phosphoric acid catalyst†

**DOI:** 10.1039/c7ra13471a

**Published:** 2018-02-12

**Authors:** Lidia Clot-Almenara, Carles Rodríguez-Escrich, Miquel A. Pericàs

**Affiliations:** Institute of Chemical Research of Catalonia (ICIQ), The Barcelona Institute of Science and Technology Av. Països Catalans 16 43007 Tarragona Spain mapericas@iciq.es; Departament de Química Inorgànica i Orgànica, Universitat de Barcelona 08080 Barcelona Spain

## Abstract

A polystyrene-supported BINOL-derived chiral phosphoric acid has been applied to the desymmetrisation of *meso*-diones to produce enantioenriched cyclohexenones. The catalytic resin has proven highly active and robust, giving rise to Hajos–Parrish or Wieland–Miescher type products in good yields and enantioselectivities, while allowing for extended recycling.

## Abstract 2

A polystyrene-supported, recyclable, BINOL-derived chiral phosphoric acid has been applied to the desymmetrization of *meso*-diones to produce enantioenriched cyclohexenones.

## Graphical abstract

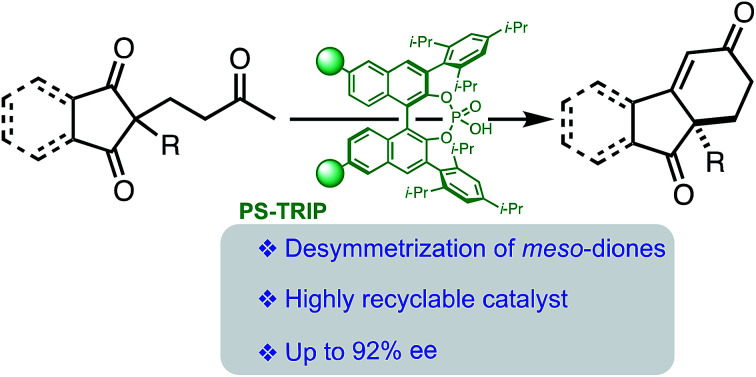

## Introduction

**Scheme 1:**
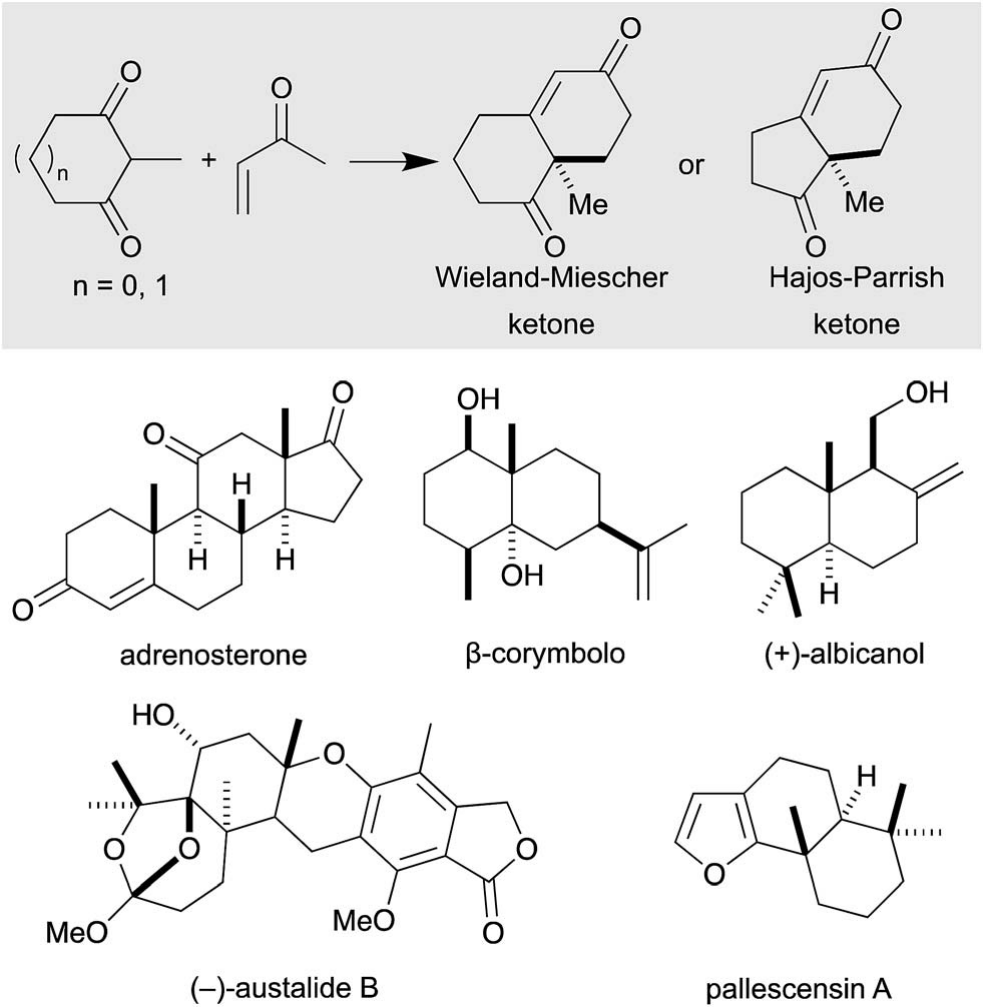
Wieland–Miescher and Hajos–Parrish ketones; natural products prepared *via* Robinson annulation.

The Robinson annulation represents one of the most powerful synthetic strategies for the synthesis of cyclohexenone derivatives,^1^ which are extremely versatile building blocks for the preparation of natural products. In 1971, two industrial research groups, Hajos and Parrish^2^ (working at Hoffmann-La Roche) and Eder, Sauer and Weichert^3^ (from Schering AG), independently discovered a catalytic asymmetric variant of the Robinson annulation that was mediated by proline, in what is nowadays considered one of the most important breakthroughs in organocatalysis.^4^ This transformation, also known as the Hajos–Parrish–Eder–Sauer–Wiechert reaction (Scheme 1, top), has emerged as a powerful tool for the total synthesis of natural products^5^ and bioactive compounds,^6^ predominantly sesquiterpenoids,^7^ terpenoids^8^ and steroids,^9^ which stand out for their antimicrobial, antiviral and anticancer effect (Scheme 1, bottom).

Efforts to identify an alternative organocatalyst able to promote the asymmetric Robinson annulation include proline derivatives,^10^ amines,^11^ amino acids,^12^ peptides,^13^ antibodies,^14^ prolinamide^15^ or chiral vicinal diamines.^16^ It is thus evident that the common motif in most of the catalytic entities employed so far is the presence of a chiral amine, which is assumed to promote the reaction through the enamine activation mode.^17^ However, according to the commonly accepted mechanistic rationalisations, the presence of a base slows down the second half of the process, namely, the dehydration of the β-hydroxyketone intermediate. This step is accelerated in acidic media and high temperatures, which is why several protocols involve addition of an acid after the starting material is consumed. Actually, even Brønsted acids alone are known to promote the Robinson annulation in an efficient manner.^1*b*^

On the basis of these precedents, Akiyama and co-workers hypothesised that chiral phosphoric acids (CPA) should be able to mediate the desymmetrisation of *meso*-1,3-diones in the absence of an acidic additive; indeed, the proof of concept was reported in 2009.^18^ In this case, in contrast to what happens in the aminocatalytic processes, the stereoselectivity was controlled *via* non-covalent interactions and the same catalyst promoted the desymmetrisation and the final dehydration.

**Fig. 1:**
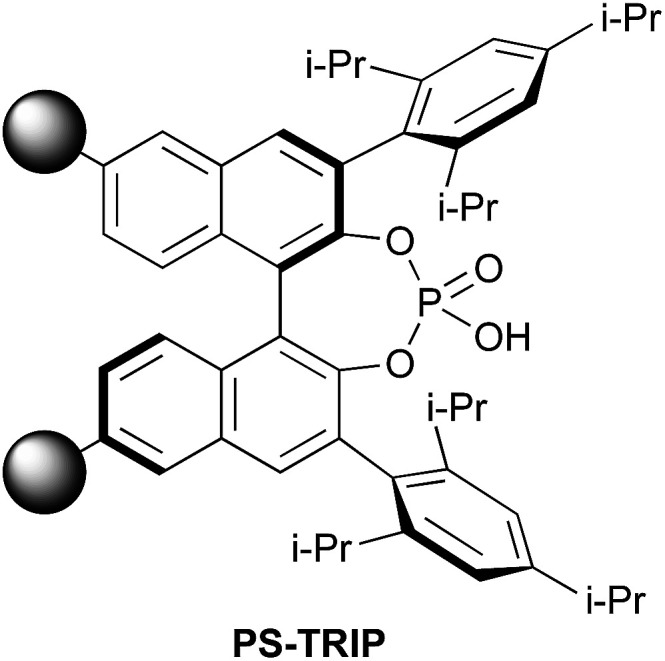
Polystyrene-supported TRIP phosphoric acid catalyst.

Chiral phosphoric acids were independently developed in 2004 by Akiyama and Terada^19^ in an attempt to harness the reactivity of imines. However, these Brønsted acid catalysts have proven to be much more versatile than initially expected, giving high levels of enantioinduction in very disparate reactions.^20^ Perhaps the main drawback of these CPAs is the long and tedious reaction sequences required for their preparation. In this scenario, catalyst immobilisation^21^ can provide a solution by rendering materials that ideally retain the properties of the monomer while being insoluble in the reaction media. With this idea in mind, several research groups, including our own, have studied the immobilisation of CPAs to assess the impact of this approach on the activity and recyclability of the resulting catalytic materials.^22^ Indeed, solid-supported CPAs^23^ turned out to be very active and robust; remarkably, in the event of loss of catalytic activity, simply washing the catalytic resin with acid restored its initial behaviour. More recently, we turned our attention to the CPA that has been more successful to date, namely, the TRIP catalyst developed by List *et al.*^24^ Thus, we prepared PS-TRIP (Fig. 1) based on a co-polymerisation strategy and applied it to the asymmetric allylboration of aldehydes.^25^ The catalytic resin admitted recycling in batch and the implementation of a flow process spanning 28 h. It is worth noting that, about the same time, Kobayashi *et al.* reported on a very similar immobilisation strategy to obtain a hybrid material that combined supported TRIP and Au/Pd nanoparticles.^26^ This heterogeneous catalyst exploited the benefits of both catalysts, allowing to carry out a sequence of oxidation and enantioselective *aza*-Friedel–Crafts reaction.

**Fig. 2:**
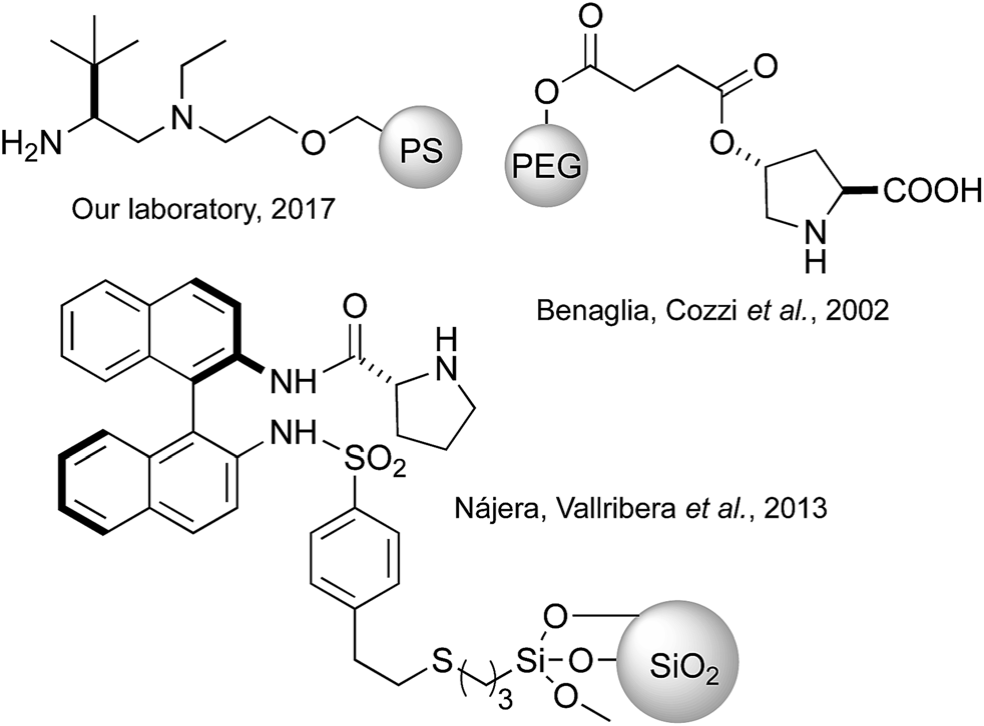
Selection of previously reported immobilised catalysts for the desymmetrisation of *meso*-diones.

These previous results encouraged us to tackle the applicability of the PS-TRIP catalyst to the desymmetrisation of *meso*-1,3-diones to produce chiral cyclohexenones, with particular interest in the easy separation of final product and catalyst and the recovery of the latter. As previously mentioned, the acidic nature of the catalyst enables the application of the same reaction conditions to carry out both the desymmetrisation and the dehydration, without having to use any additive or modify the temperature. In contrast, previous attempts to apply solid-supported catalysts to promote this reaction have relied in aminocatalytic approaches involving proline or prolinamide derivatives supported on polystyrene,^27^ silica^15,28^ or polyethyleneglycol (Fig. 2).^29^ More recently, the Luo diamine catalyst^16^ has been supported onto polystyrene, proving to be recyclable and allowing operation in continuous flow.^30^

## Results and discussion

On the basis of the homogeneous precedent,^18^ we decided to initiate our investigation using 1a as a model substrate with our PS-TRIP catalyst in different solvents (Table 1, entries 1–5). As expected, almost no reactivity was observed at room temperature with the exception of toluene and hexane (35% yield). Heating up the reaction mixture to 70 °C effectively increased the yield while maintaining high enantioselectivities (entry 6). Increasing the catalyst loading to 10 mol%, higher yields were achieved (entry 7). After these preliminary results, we decided to expand the solvent screening (entries 8–9), but toluene and DCE did not improve the results observed with hexane. Finally, we increased the catalyst loading and the reaction time (entries 10–12), establishing the optimised reaction conditions to obtain the cyclohexenone product as follows: 20 mol% catalyst, 70 °C and 48 h (98% yield, 89% ee).

**Table 1:** Screening of reaction conditions for the desymmetrisation of *meso*-dione 1a^a^

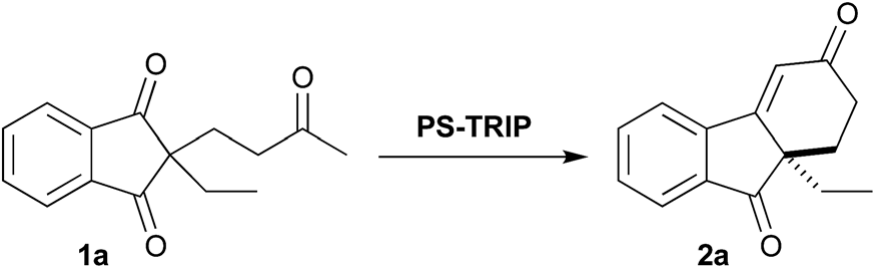						
Entry	Solvent	Temp [°C]	Cat. loading [%]	Time [h]	Yield^b^ [%]	ee^c^ [%]
1	Hexane	rt	5	32	35	90
2	EtOAc	rt	5	32	—	—
3	CH_2_Cl_2_	rt	5	32	—	—
4	THF	rt	5	32	—	—
5	Toluene	rt	5	32	Traces	—
6	Hexane	70	5	48	50	91
7	Hexane	70	10	24	80	89
8	DCE	70	10	24	64	90
9	Hexane	70	10	24	75	88
10	Hexane	70	15	24	64	88
11	Hexane	70	20	24	69	88
12	Hexane	70	20	48	98	89

a Reactions were carried out with 0.2 mmol of 1a in 2 mL of solvent.

b Yield of isolated product.

c Determined by HPLC on a chiral stationary phase.

**Scheme 2:**
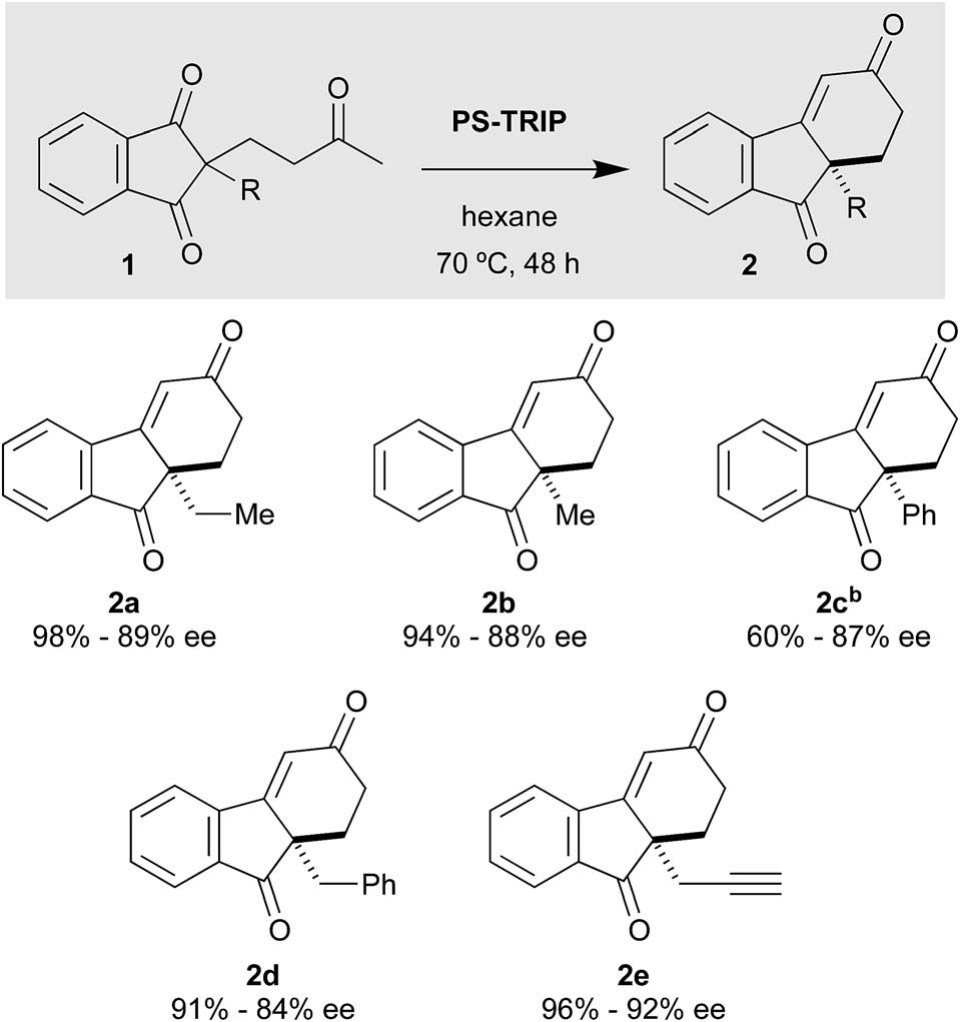
Scope of the desymmetrisation reaction with benzene-fused *meso*-diones 1.^a a^ Unless otherwise stated, the reactions were carried out with 0.12 mmol of 1, 20 mol% of PS-TRIP in 1.2 mL of hexane. ^b^ In toluene at 90 °C.

With the optimised conditions in hand, we moved to explore the reaction scope, starting with the benzo-fused *meso*-diones (Scheme 2). The methyl-substituted analogue 2b was obtained in excellent yield and enantioselectivity (94%, 88% ee).

The compound bearing a phenyl group gave moderate yields, which can be attributed to the low solubility of the starting material. Indeed, when the reaction was carried out in toluene at 90 °C, 2c was obtained in good yields. In comparison, the benzyl-substituted compound gave rise to the cyclohexenone 2d in considerably higher yields. As for the propargylic substrate, it displayed an excellent behaviour, furnishing 2e (an intermediate used in the synthesis of a gibbane framework^2*d*^) in excellent yield and ee. In general, we can say that this type of substrates give rise to the desired cyclohexenones in very good yields and enantioselectivities, despite the somewhat elevated reaction temperatures employed.

**Scheme 3:**
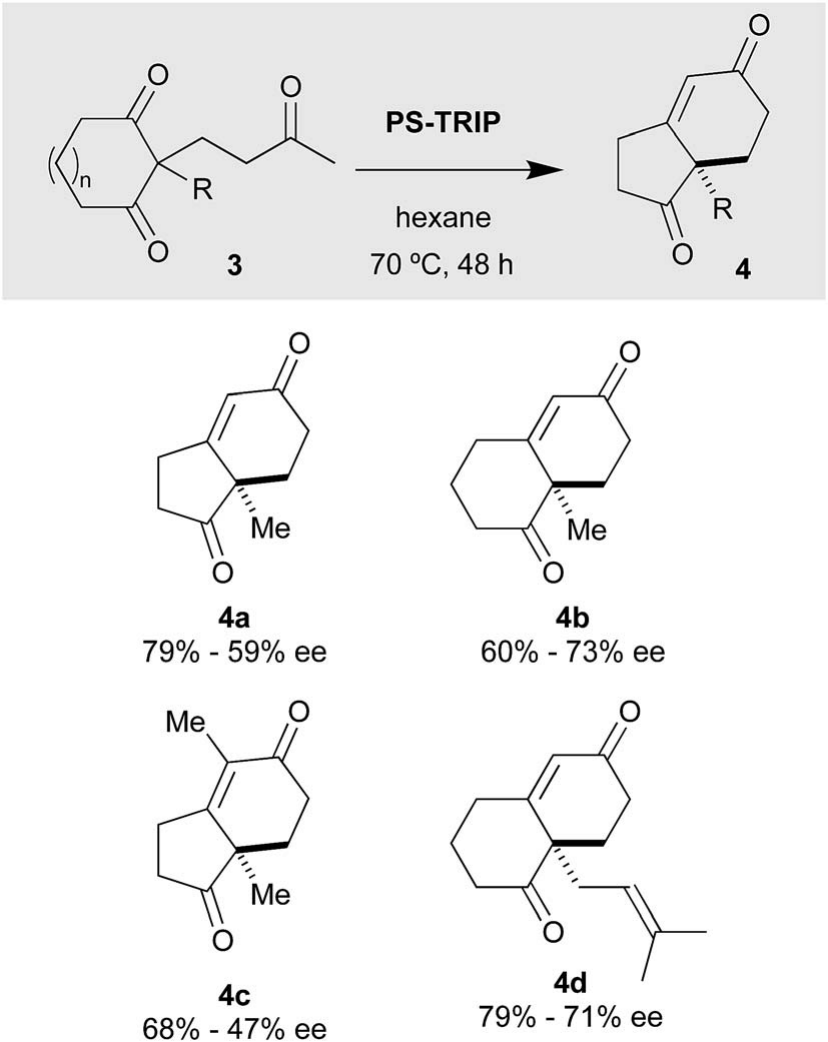
Scope of the desymmetrisation reaction with cyclic *meso*-diones 3.^a a^ Reactions were carried out with 0.12 mmol of 3, 20 mol% of PS-TRIP in 1.2 mL of hexane.

The results obtained with *meso*-diones 3, lacking the fused benzene ring, are summarised in Scheme 3; in this case the results were not as good as those recorded for the tricyclic compounds. For instance, the Hajos–Parrish 4a and Wieland–Miescher 4b ketones were synthesised in decent yields but moderate ee's. Our PS-TRIP catalyst was also able to give rise to 4c, bearing a tetrasubstituted alkene moiety, in 68% yield, but unfortunately in low enantioselectivity. Higher yield was observed in the case of the synthesis of Wieland–Miescher ketone derivative 4d, bearing the classical dimethylallyl group present in many terpene derivatives.

The PS-TRIP resin has shown not only to be active in a wide variety of substrates, but also to be highly recyclable. Remarkably, all substrates have been run with two samples of polymer, which were already employed in the screening table. Accordingly, each sample of catalyst has been used 9 times in total without appreciable decay in activity. After each run, the catalyst was rinsed, dried in the vacuum line overnight and reused again without further reconditioning. Considering the multi-step, tedious preparation of the TRIP catalyst, this feature allows saving huge amounts of solvent and chemical waste, thus contributing to the greenness of the overall process.

## Conclusions

In summary, we have established an efficient strategy for the desymmetrisation of *meso*-1,3-diones catalysed by an immobilised version of the TRIP phosphoric acid catalyst. This approach can be applied to a broad range of starting *meso*-diones, giving rise to the corresponding cyclised products in up to 98% yield and 92% enantiomeric excess. In this work, we also demonstrate the efficiency of the PS-TRIP catalyst, which is easily recovered by simple filtration and reused at least nine times, thus facilitating the isolation of the final product.

## Conflicts of interest

There are no conflicts to declare.

## Supplementary Material

RA-008-C7RA13471A-s001
