# Supplementary material for: Desymmetrisation of meso-diones promoted by a highly recyclable polymer-supported chiral phosphoric acid catalyst
Source: RSC Adv. 2018 Feb 12;8(13):6910–4. doi: 10.1039/c7ra13471a (PMC9078317; doi:10.1039/c7ra13471a)
Supplement: RA-008-C7RA13471A-s001 [file RA-008-C7RA13471A-s001.pdf]

Electronic Supplementary Material (ESI) for RSC Advances.
This journal is © The Royal Society of Chemistry 2018

Supporting Information

Desymmetrisation of *meso*-diones promoted by a highly recyclable polymer-supported chiral phosphoric acid catalyst

Lidia Clot-Almenara,[a] Carles Rodríguez-Escrich,*[,a] and Miquel A. Pericàs*[,a,b]

crodriguez@iciq.es, mapericas@iciq.es

[a] Institute of Chemical Research of Catalonia (ICIQ), The Barcelona Institute of Science and Technology, Av. Països Catalans, 16, 43007 Tarragona (Spain)
and
[b] Departament de Química Inorgànica i Orgànica,
Universitat de Barcelona (UB), 08028 Barcelona (Spain)

Table of Contents

1. General Information S2

2. Synthesis of the Polystyrene-Supported Chiral Phosphoric Acid (**PS-TRIP**) S3

3. General Procedure for the Desymmetrization of *meso*-1,3-Diones S5

4. Characterization of the Cyclohexenone Products S6

5. ^{1}H and ^{13}C NMR Spectra S9

6. HPLC Chromatograms S19

1. General Information

All reactions utilizing air- and moisture-sensitive reagents were carried out under a dry Argon atmosphere in oven-dried material. All solvents used in the reactions were dried using an SPS (Solvent Purification System) unless otherwise stated. Thin layer chromatography was performed on Merck TLC Silicagel 60 F254 aluminium sheets. Components were visualized by UV light (λ = 254 nm) and stained with *p*-anisaldehyde or phosphomolybdic dip. Flash column chromatography was carried out using Sigma-Aldrich 60 mesh silica gel and dry-packed columns. ^{1}H NMR and ^{13}C NMR spectra were recorded at 298 K on a Bruker Avance 500 or 400 Ultrashield apparatus. ^{1}H NMR spectroscopy chemical shifts are quoted in ppm relative to tetramethylsilane (TMS). $CDCl_3$ was used as internal standard for ^{13}C NMR spectra. Chemical shifts are given in δ and coupling constants in Hz. IR spectra were recorded on a Bruker Tensor 27 FT-IR spectrometer and are reported in wavenumbers (cm^{-1}). Elemental analyses were performed by MEDAC Ltd. (Surrey, UK) on a LECO CHNS 932 micro-analyzer. High performance liquid chromatography (HPLC) was performed on Agilent Technologies chromatographs (1100 and 1200 Series), using Chiralcel or Chiralpak columns and guard column. The column employed in each case is indicated. Racemic standard products were prepared according to the reported procedure using DL-Proline as catalyst[1]. High resolution mass spectrometry analyses were performed in a Waters LCD Premier™ instrument operating in ESI (Electro-Spray Ionization) mode or APCI (Atmospheric-Pressure Chemical Ionization) mode. Specific optical rotation measurements were carried out on a Jasco P-1030 polarimeter.

2. Synthesis of the Polystyrene-Supported Chiral Phosphoric Acid (PS-TRIP)

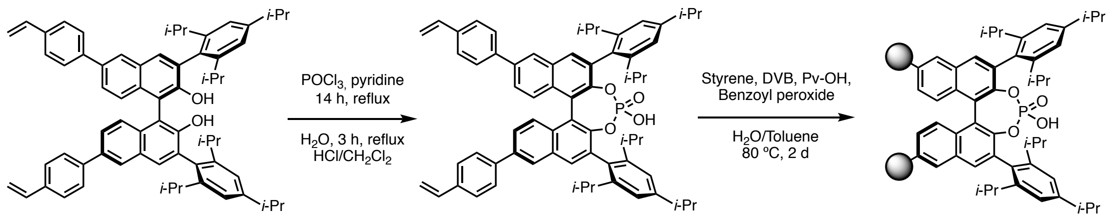

5 **6** **PS-TRIP**

The procedure is adapted from our previous paper[2] with the slight modifications noted below.

Modified (*R*)-TRIP phosphoric acid (6)[3]

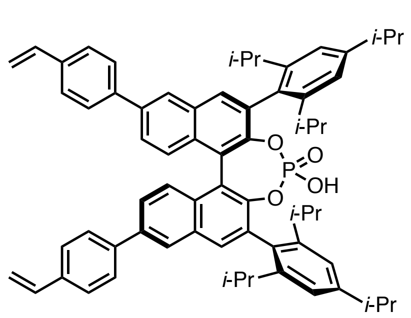

6

A flame-dried Schlenk tube, was charged with a solution of BINOL derivative **5**[2] (1 g, 1.12 mmol) in pyridine (2.3 mL) under Ar. Then, $POCl_3$ (0.31 mL, 3.35 mmol, 53 equiv.) was added, and the reaction mixture was heated in the closed Schlenk tube for 14 h at 120 °C. After this time, the reaction was allowed to reach RT, followed by addition of water (2.3 mL). The resulting brownish slurry was heated to reflux for 3 h. Once the reaction was completed, the solution was cooled to RT, 10 mL of CH_2Cl_2 were added and the resulting organic phase was thoroughly washed with 1 M HCl (3 × 5 mL). The resulting organic layers were dried over $MgSO_4$ and recrystallized from ACN. The aqueous solution should have a pH = 1–2 to ensure **6** is in the acid from.

^{1}H NMR (400 MHz, $CDCl_3$): δ 8.07 (d, *J* = 1.9 Hz, 2H), 7.87 (s, 2H), 7.70 (d, *J* = 8.3 Hz, 4H), 7.62 (dd, *J* = 8.9, 1.9 Hz, 2H), 7.54 (d, *J* = 8.3 Hz, 4H), 7.45 (d, *J* = 8.9 Hz, 2H), 6.95 (s, 4H), 6.78 (dd, *J* = 17.6, 10.9 Hz, 2H), 5.82 (dd, *J* = 17.6, 0.9 Hz, 2H), 5.30 (dd, *J* = 10.9, 0.9 Hz, 2H), 2.91 – 2.78 (m, 2H), 2.67 – 2.54 (m, 4H), 1.23 (dd, *J* = 6.9, 4.8 Hz, 12H), 1.08 (d, *J* = 6.7 Hz, 6H), 1.01 (d, *J* = 6.8 Hz, 6H), 0.93 (d, *J* = 6.8 Hz, 6H), 0.80 (d, *J* = 6.7 Hz, 6H).

^{13}C NMR (126 MHz, $CDCl_3$): δ ^{13}C NMR (126 MHz, $CDCl_3$) δ 148.6 (×2), 148.1 (×2), 147.6 (×2), 140.1 (×2), 138.2 (×2), 137.1 (×2), 136.5 (×2), 133.0 (×2), 132.9 (×2), 131.7 (×2), 131.5 (×2), 131.4 (×2), 128.1 (×2), 127.6 (×4), 127.0 (×4), 126.0 (×2), 125.8 (×2), 122.1 (×2), 122.1 (×2), 121.3 (×2), 120.4 (×2), 114.3 (×2), 34.4 (×2), 31.1 (×2), 30.8 (×2), 26.5 (×2), 25.2 (×2), 24.3 (×2), 24.0 (×2), 23.4 (×2), 22.9 (×2).

$[\alpha]_D$: −1.4 (*c* 1.00, CH_2Cl_2).

Polystyrene Supported (*R*)-TRIP (PS-TRIP)

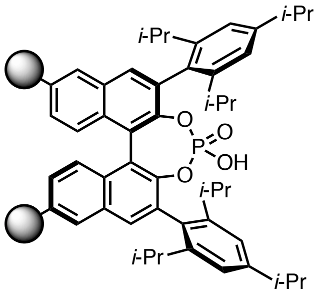

PS-TRIP

A 100 mL reactor was charged with a suspension of polyvinyl alcohol (PV-OH) (38 mg, 0.37 µmol, 0.001 equiv.) in 38 mL of deionized and degassed water. The solution was heated at 90 °C until PV-OH was dissolved. Then, it was cooled to RT and a solution under N_2, containing divinylbenzene (DVB; 80%, 111 µL, 0.62 mmol, 1.7 equiv.) filtered on a short pad of silica immediately before use, phosphoric acid **6** (350 mg, 0.37 mmol, 1 equiv.), styrene (2.98 mL, 25.9 mmol, 70.8 equiv.) and benzoyl peroxide (75%, 39 mg, 0.12 mmol, 0.33 equiv.) in toluene (1.27 mL) was transferred to the reactor. After that, the system was heated at 80 °C and stirred at 440 rpm. After two days, the aqueous solution was decanted off and the resin was washed with water (50 °C) several times, followed by THF, EtOAc, 2 M HCl/EtOAc, and CH_2Cl_2. Finally, it was dried overnight in a vacuum oven at 40 °C.

P elemental analysis (%): 0.39.

$\boldsymbol{f_{(P)}}$: 0.13 mmol/$g_{resin.}$

3. General Procedure for the Desymmetrization of *meso*-1,3-Diones

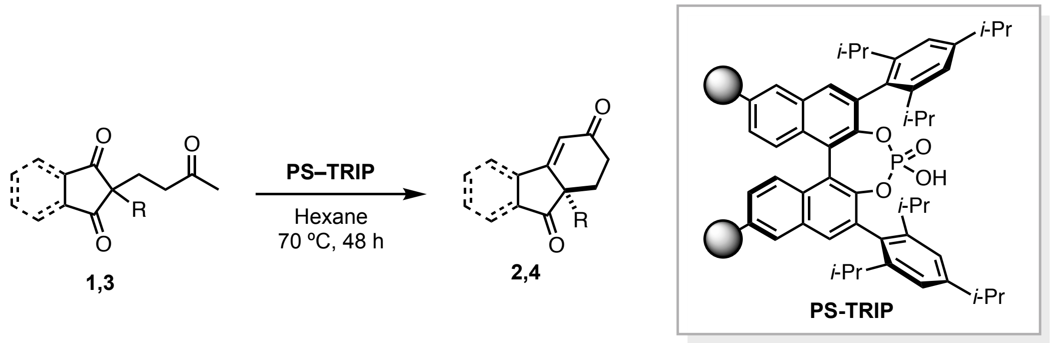

PS-TRIP (20 mol%) was added at room temperature to a screw-cap reaction tube containing the *meso*-dione (0.12 mmol) in hexane (1.2 mL). Then, the reaction was heated at 70 °C, and monitored by TLC. After 48 h, the resin was filtered and rinsed with CH_2Cl_2. The filtrate was directly loaded on a silica gel column and the crude product was purified by flash chromatography using cyclohexane/EtOAc.

4. Characterization of the Cyclohexenone Products

(*R*)-9a-Ethyl-1,9a-dihydro-3*H*-fluorene-3,9(2*H*)-dione (**2a**)[4]

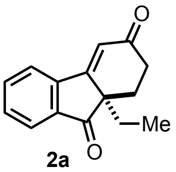

2a

Compound **2a** was obtained in 98% yield and 89% ee as a slightly yellow solid. **^{1}H NMR** (400 MHz, CDCl3): δ 7.85 (dt, *J* = 7.6, 1.0 Hz, 1H), 7.80 (dt, *J* = 7.8, 1.0 Hz, 1H), 7.74 (td, *J* = 7.5, 1.2 Hz, 1H), 7.63 (td, *J* = 7.4, 1.1 Hz, 1H), 6.36 (s, 1H), 2.69 (ddd, *J* = 18.9, 13.5, 5.5 Hz, 1H), 2.61 – 2.52 (m, 1H), 2.42 (ddd, *J* = 13.5, 5.5, 1.8 Hz, 1H), 2.00 – 1.8 (m, 2H), 1.87-1.75 (m, 1H), 0.81 (t, *J* = 7.5 Hz, 3H). **^{13}C NMR** (101 MHz, CDCl3): δ 203.9, 198.8, 162.4, 145.3, 137.0, 135.5, 132.6, 124.3, 123.0, 118.6, 52.0, 33.6, 29.1, 25.6, 9.6. **HPLC** (Daicel Chiralpak IB column, hexane/*i*-PrOH 80:20, flow rate 1.0 mL/min, λ = 250 nm): t_{major} = 16.0 min.; t_{minor} = 15.1 min.

(*R*)-9a-Methyl-1,9a-dihydro-3*H*-fluorene-3,9(2*H*)-dione (2b)[4]

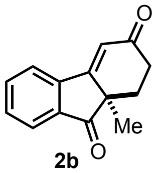

2b

Compound **2b** was obtained in 94% yield and 88% ee as white solid. **^{1}H NMR** (400 MHz, $CDCl_3$): δ 7.87 (dt, *J* = 7.6, 1.1 Hz, 1H), 7.81 (dt, *J* = 7.7, 1.0 Hz, 1H), 7.75 (ddd, *J* = 7.8, 7.1, 1.1 Hz, 1H), 7.64 (td, *J* = 7.4, 1.1 Hz, 1H), 6.36 (s, 1H), 2.79 – 2.68 (m, 1H), 2.66 – 2.56 (m, 1H), 2.30 (ddd, *J* = 13.2, 5.3, 1.8 Hz, 1H), 2.01 (td, *J* = 13.4, 5.6 Hz, 1H), 1.42 (s, 3H). **^{13}C NMR** (126 MHz, $CDCl_3$): δ 204.2, 198.7, 162.3, 144.6, 135.9, 135.6, 132.6, 124.9, 123.5, 118.7, 48.4, 33.8, 27.7, 22.4. **HPLC** (Daicel Chiralpak OD-H column, hexane/*i*-PrOH 83:17, flow rate 0.5 mL/min, λ = 300 nm): t_{major} = 22.7 min.; t_{minor} = 20.3 min.

(*S*)-9a-Phenyl-1,9a-dihydro-3*H*-fluorene-3,9(2*H*)-dione (2c)[4]

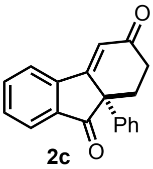

2c

Compound **2c** was obtained in 60% yield and 87% ee as a white solid. **^{1}H NMR** (500 MHz, $CDCl_3$): δ 7.93 (dt, *J* = 7.7, 0.9 Hz, 1H), 7.83 – 7.72 (m, 2H), 7.61 (td, *J* = 7.5, 1.0 Hz, 1H), 7.55 – 7.47 (m, 2H), 7.35 – 7.22 (m, 3H), 6.67 (s, 1H), 2.82 (ddd, *J* = 12.7, 4.5, 2.2 Hz, 1H), 2.49 (m, 1H), 2.37 – 2.16 (m, 2H). **^{13}C NMR** (126 MHz, $CDCl_3$): δ 200.7, 199.2, 159.7, 145.5, 136.9, 136.2, 135.8, 132.9, 129.1 (×2), 128.2, 127.5 (×2), 125.2, 123.1, 120.9, 57.5, 34.4, 30.9. **HPLC** (Daicel Chiralpak AD-H column, hexane/*i*-PrOH 90:10, flow rate 0.6 mL/min, λ = 240 nm): t_{major} = 28.6 min.; t_{minor} = 27.3 min.

(*S*)-9a-Benzyl-1,9a-dihydro-3*H*-fluorene-3,9(2*H*)-dione (2d)[4]

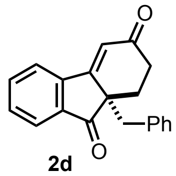

2d

Compound **2d** was obtained in 91% yield and 84% ee as a slightly yellow solid. **^{1}H NMR** (400 MHz, $CDCl_3$): δ 7.63 (dt, *J* = 7.6, 1.0 Hz, 1H), 7.59 – 7.51 (m, 2H), 7.49 – 7.38 (m, 1H), 6.99 (dd, *J* = 5.0, 1.9 Hz, 3H), 6.92 – 6.82 (m, 2H), 6.43 (s, 1H), 3.20 (d, *J* = 13.0 Hz, 1H), 3.14 (d, *J* = 13.1 Hz, 1H), 2.83 (ddd, *J* = 19.1, 13.5, 5.7 Hz, 1H), 2.71 – 2.56 (m, 1H), 2.44 (ddd, *J* = 13.5, 5.8, 1.6 Hz, 1H), 2.05 (td, *J* = 13.6, 5.9 Hz, 1H). **^{13}C NMR** (101 MHz, $CDCl_3$): δ 203.7, 198.6, 161.3, 145.7, 137.3, 135.2, 135.2, 132.2, 129.7 (×2), 128.0 (×2), 127.0, 123.8, 122.6, 119.3, 53.7, 42.8, 33.8, 27.2. **HPLC** (Daicel Chiralpak AD-H column, hexane/*i*-PrOH 90:10, flow rate 0.5 mL/min, λ = 300 nm): t_{major} = 28.4 min.; t_{minor} = 25.4 min.

(*S*)-9a-(Prop-2-yn-1-yl)-1,9a-dihydro-3*H*-fluorene-3,9(2*H*)-dione (2e)[4]

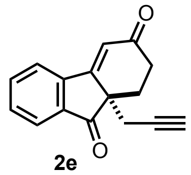

2e

Compound **2e** was obtained in 96% yield and 92% ee as a slightly yellow solid. **^{1}H NMR** (500 MHz, $CDCl_3$): δ 7.88 (dt, *J* = 7.7, 1.0 Hz, 1H), 7.81 (dt, *J* = 7.7, 1.0 Hz, 1H), 7.75 (td, *J* = 7.5, 1.2 Hz, 1H), 7.64 (td, *J* = 7.4, 1.1 Hz, 1H), 6.42 (s, 1H), 2.82 – 2.74 (m, 1H), 2.72 (d, *J* = 2.8 Hz, 1H), 2.69 (d, *J* = 2.7 Hz, 1H), 2.67 – 2.62 (m, 1H), 2.61 – 2.53 (m, 1H), 2.03 (td, *J* = 13.6, 5.8 Hz, 1H), 1.96 (t, *J* = 2.7 Hz, 1H). **^{13}C NMR** (126 MHz, $CDCl_3$): δ 201.8, 198.1, 159.7, 145.2, 136.4, 135.8, 132.7, 124.7, 123.1, 119.6, 78.4, 73.5, 50.5, 33.6, 26.3, 25.9.

HPLC (Daicel Chiralpak AD-H column, hexane/*i*-PrOH 90:10, flow rate 1.0 mL/min, λ = 300 nm): t_{major} = 20.2 min.; t_{minor} = 18.9 min.

(*R*)-7a-Methyl-2,3,7,7a-tetrahydro-1*H*-indene-1,5(6*H*)-dione (4a)[4]

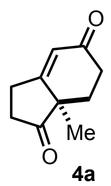

4a

Compound **4a** was obtained in 79% yield and 59% ee as a slightly yellow oil. **^{1}H NMR** (500 MHz, $CDCl_3$): δ 5.97 (br d, *J* = 2.5 Hz, 1H), 3.06 – 2.90 (m, 1H), 2.84 – 2.70 (m, 2H), 2.58 – 2.38 (m, 3H), 2.11 (ddd, *J* = 13.6, 5.2, 2.2 Hz, 1H), 1.91 – 1.80 (m, 1H), 1.32 (s, 3H). **^{13}C NMR** (126 MHz, $CDCl_3$): δ 216.6, 198.3, 169.8, 124.1, 48.9, 36.0, 33.1, 29.4, 27.0, 20.7. **HPLC** (Daicel Chiralpak AD-H column, hexane/*i*-PrOH 95:5, flow rate 1.0 mL/min, λ = 240 nm): t_{major} = 18.2 min.; t_{minor} = 17.2 min.

(*R*)-8a-Methyl-3,4,8,8a-tetrahydronaphthalene-1,6(2*H*,7*H*)-dione (4b)[4]

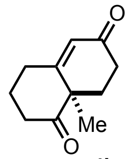

4b

Compound **4b** was obtained in 60% yield and 73% ee as a colourless oil. **^{1}H NMR** (400 MHz, $CDCl_3$): δ 5.86 (d, *J* = 1.9 Hz, 1H), 2.78 – 2.66 (m, 2H), 2.54 – 2.37 (m, 4H), 2.20 – 2.07 (m, 3H), 1.78 – 1.68 (m, 1H), 1.45 (s, 3H). **^{13}C NMR** (126 MHz, $CDCl_3$): δ 126.0, 50.8, 37.8, 33.8, 31.9, 29.9 (×2), 23.4, 23.1, 1.2 (×2). **HPLC** (Daicel Chiralpak IC column, hexane/*i*-PrOH 80:20, flow rate 1.0 mL/min, λ = 250 nm): t_{major} = 29.0 min.; t_{minor} = 33.5 min.

(*R*)-4,7a-Dimethyl-2,3,7,7a-tetrahydro-1*H*-indene-1,5(6*H*)-dione (4c)[5]

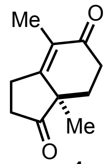

4c

Compound **4c** was obtained in 68% yield and 47% ee as a pale yellow oil. **^{1}H NMR** (500 MHz, $CDCl_3$): δ 2.94 – 2.86 (m, 1H), 2.83 – 2.72 (m, 2H), 2.58 – 2.37 (m, 3H), 2.05 (ddd, *J* = 13.4, 5.3, 2.1 Hz, 1H), 1.83 (td, *J* = 13.7, 5.8 Hz, 1H), 1.76 (d, *J* = 1.5 Hz, 3H), 1.27 (s, 3H). **^{13}C NMR** (126 MHz, $CDCl_3$): δ 217.7, 197.9, 162.5, 129.8, 48.9, 35.5, 32.8, 28.9, 24.5, 21.3, 10.8. **HPLC** (Daicel Chiralpak AS-H column, hexane/*i*-PrOH 90:10, flow rate 1.0 mL/min, λ = 250 nm): t_{major} = 17.9 min.; t_{minor} = 23.5 min.

(*S*)-8a-(3-Methylbut-2-en-1-yl)-3,4,8,8a-tetrahydronaphthalene-1,6(2*H*,7*H*)-dione (4d)[5]

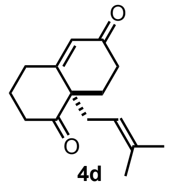

4d

Compound **5i** was obtained in 79% yield and 71% ee as a colourless oil. **^{1}H NMR** (500 MHz, $CDCl_3$): δ 5.89 (d, *J* = 1.8 Hz, 1H), 4.93 – 4.88 (m, 1H), 2.83 – 2.75 (m, 1H), 2.71 – 2.59 (m, 2H), 2.54 – 2.45 (m, 3H), 2.41 – 2.37 (m, 2H), 2.19 – 2.12 (m, 2H), 2.08 – 2.00 (m, 1H), 1.69 (s, 3H), 1.75 – 1.64 (m, 1H), 1.61 (s, 3H). **^{13}C NMR** (126 MHz, $CDCl_3$): δ 210.1, 198.6, 165.7, 136.4, 126.6, 117.4, 55.3, 38.5, 34.6, 33.8, 32.3, 26.6, 26.1, 23.6, 18.2. **HPLC** (Daicel Chiralpak IC column, hexane/*i*-PrOH 80:20, flow rate 0.7 mL/min, λ = 254 nm): t_{major} = 35.9 min.; t_{minor} = 48.8 min.

References

[1] S. G. Davies, A. J. Russell, R. L. Sheppard, A. D. Smith, J. E. Thomson, *Org. Biomol. Chem.* **2007**, *5*, 3190-3200.

[2] L. Clot-Almenara, C. Rodríguez-Escrich, L. Osorio-Planes, M. A. Pericàs, *ACS Catal.* **2016**, *6*, 7647-7651.

[3] H.-G. Cheng, J. Miguelez, H. Miyamura, W.-J. Yoo, S. Kobayashi, *Chem. Sci.* **2017**, *8*, 1356-1359.

[4] K. Mori, T. Katoh, T. Suzuki, T. Noji, M. Yamanaka, T. Akiyama, *Angew. Chem. Int. Ed.* **2009**, *48*, 9652-9654.

[5] S. Cañellas, C. Ayats, A. H. Henseler, M. A. Pericàs, *ACS Catal.* **2017**, *7*, 1383-1391.

5. ^{1}H and ^{13}C NMR Spectra

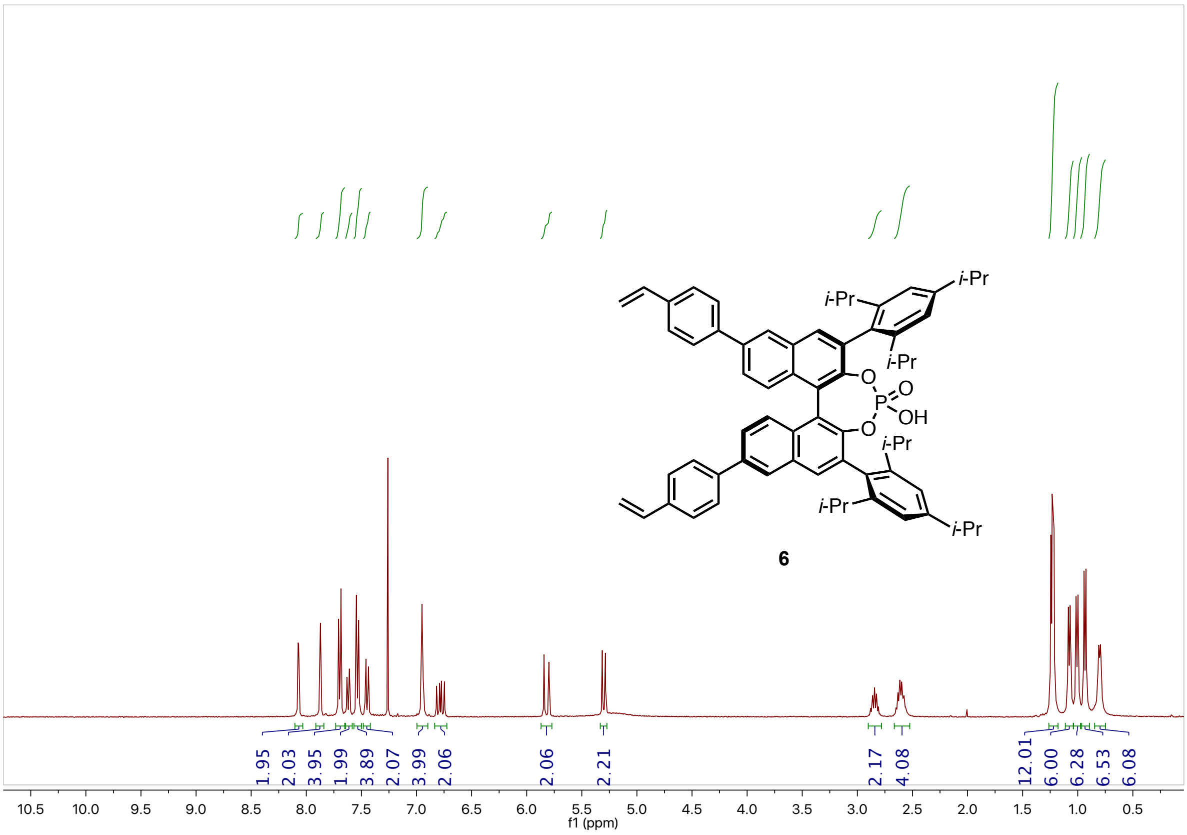

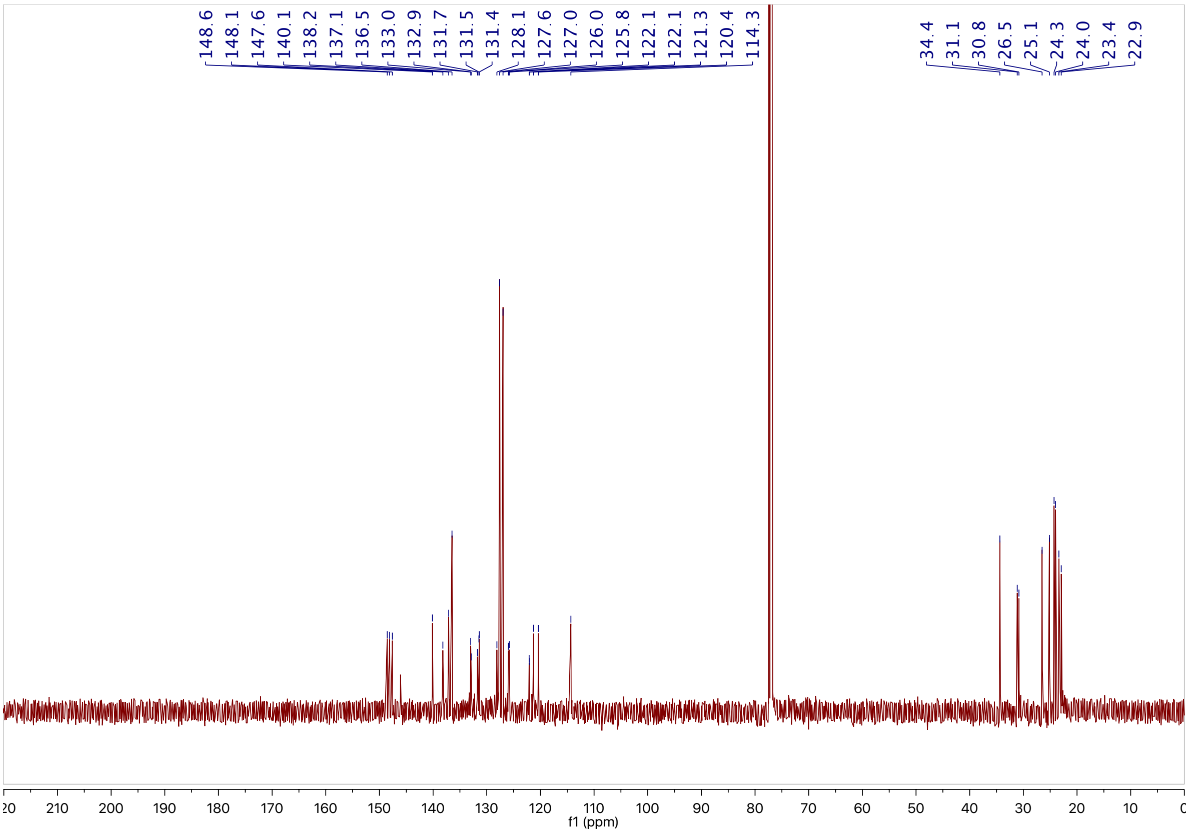

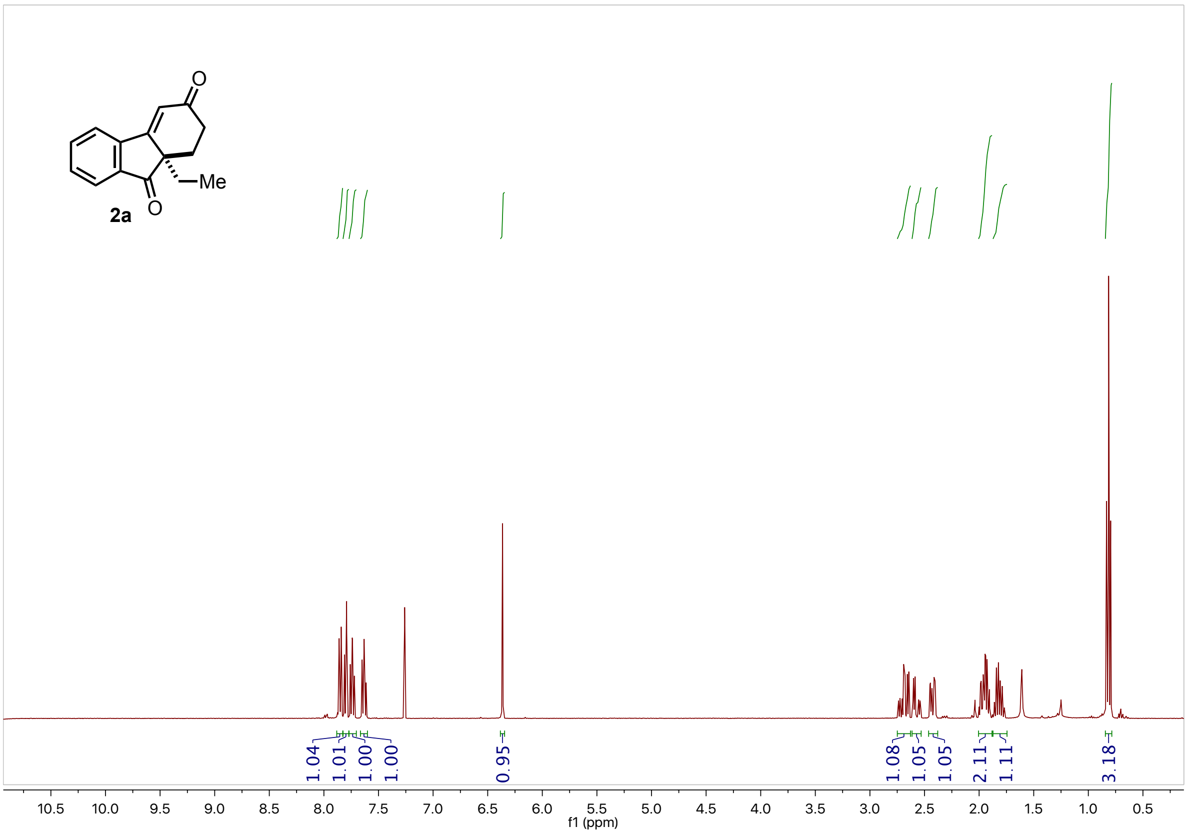
2a
Me
1.04
1.01
1.00
1.00
0.95
1.08
1.05
1.05
2.11
1.11
3.18
f1 (ppm)

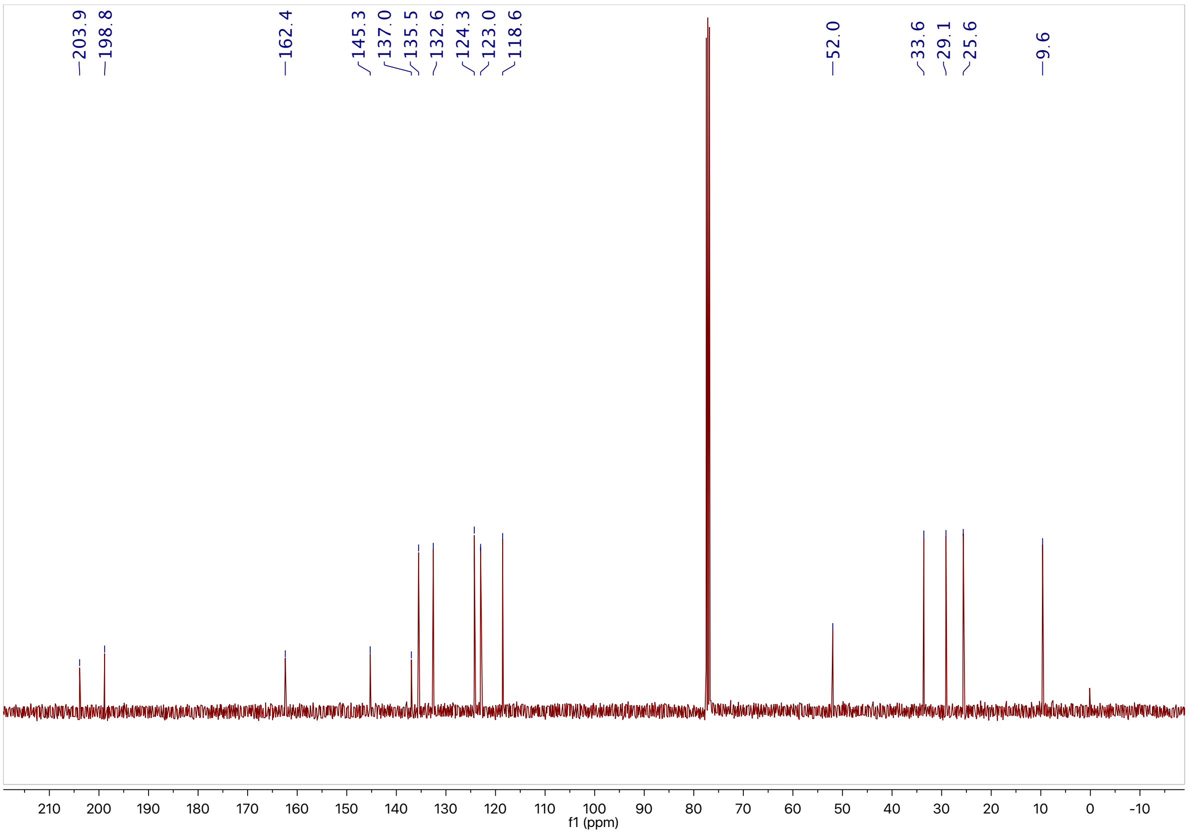
203.9
198.8
162.4
145.3
137.0
135.5
132.6
124.3
123.0
118.6
52.0
33.6
29.1
25.6
9.6
f1 (ppm)

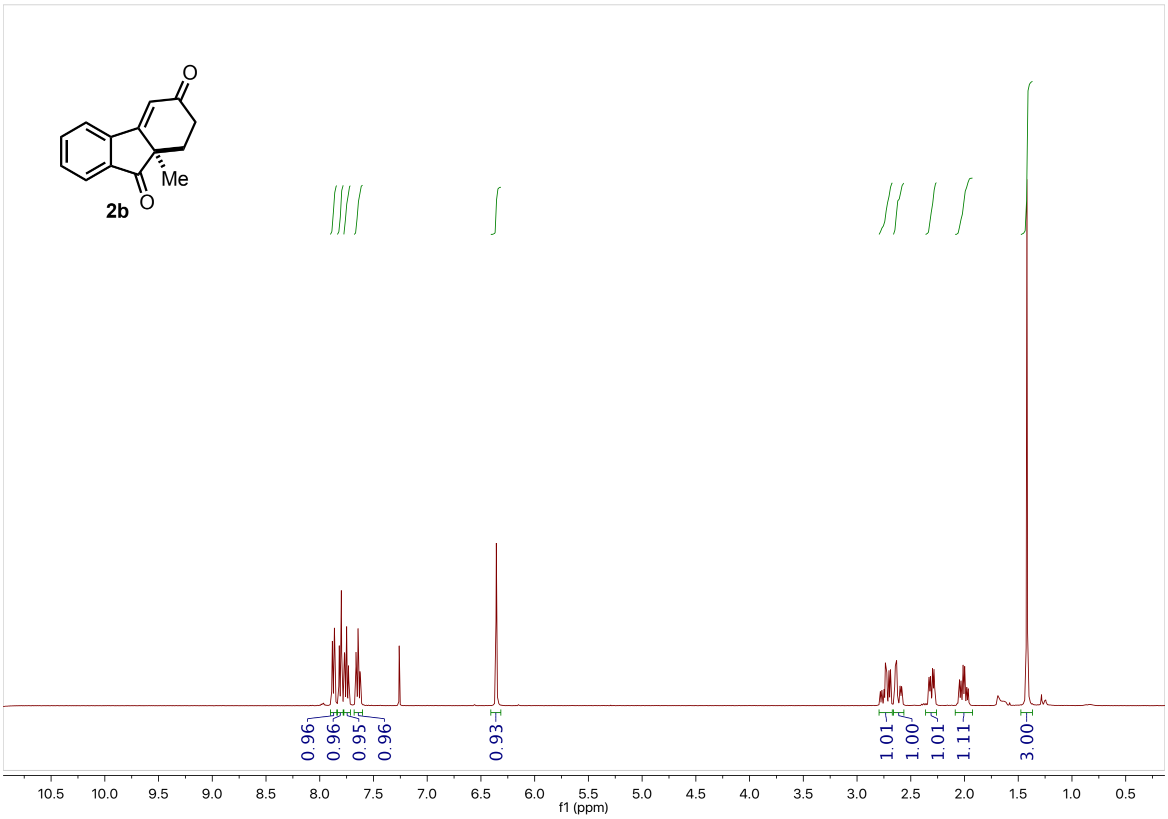
2b
Me
0.96 0.96 0.95 0.96
0.93
1.01 1.00 1.01 1.11
3.00
10.5 10.0 9.5 9.0 8.5 8.0 7.5 7.0 6.5 6.0 5.5 5.0 4.5 4.0 3.5 3.0 2.5 2.0 1.5 1.0 0.5
f1 (ppm)

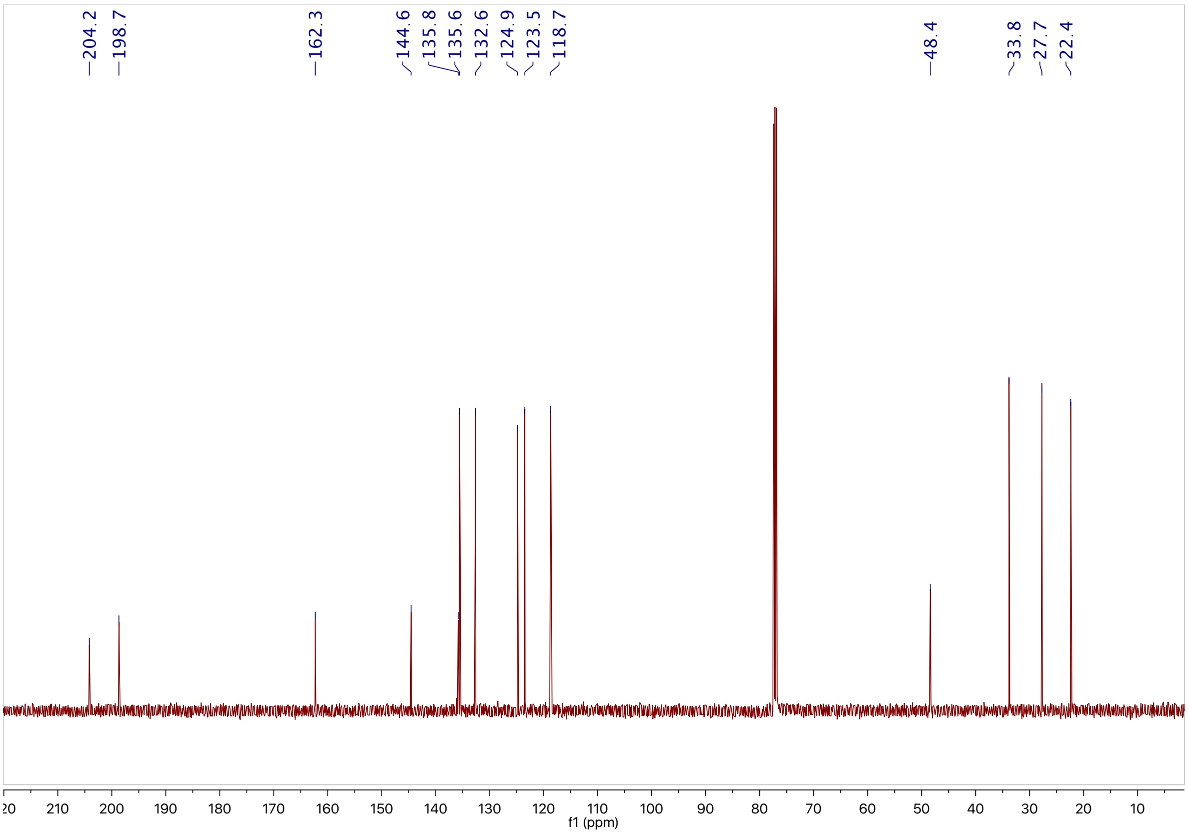
204.2
198.7
162.3
144.6
135.8
135.6
132.6
124.9
123.5
118.7
48.4
33.8
27.7
22.4
210 200 190 180 170 160 150 140 130 120 110 100 90 80 70 60 50 40 30 20 10
f1 (ppm)

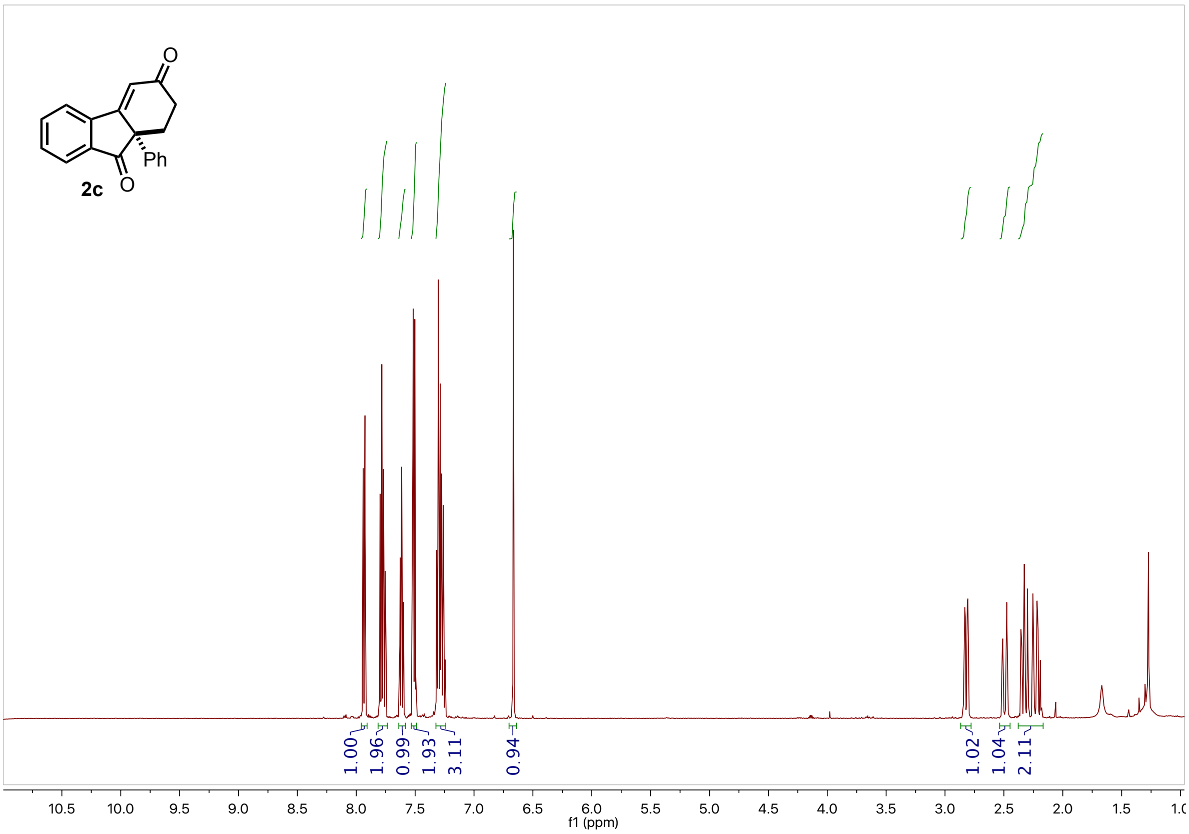
2c
Ph
1.00
1.96
0.99
1.93
3.11
0.94
1.02
1.04
2.11
f1 (ppm)

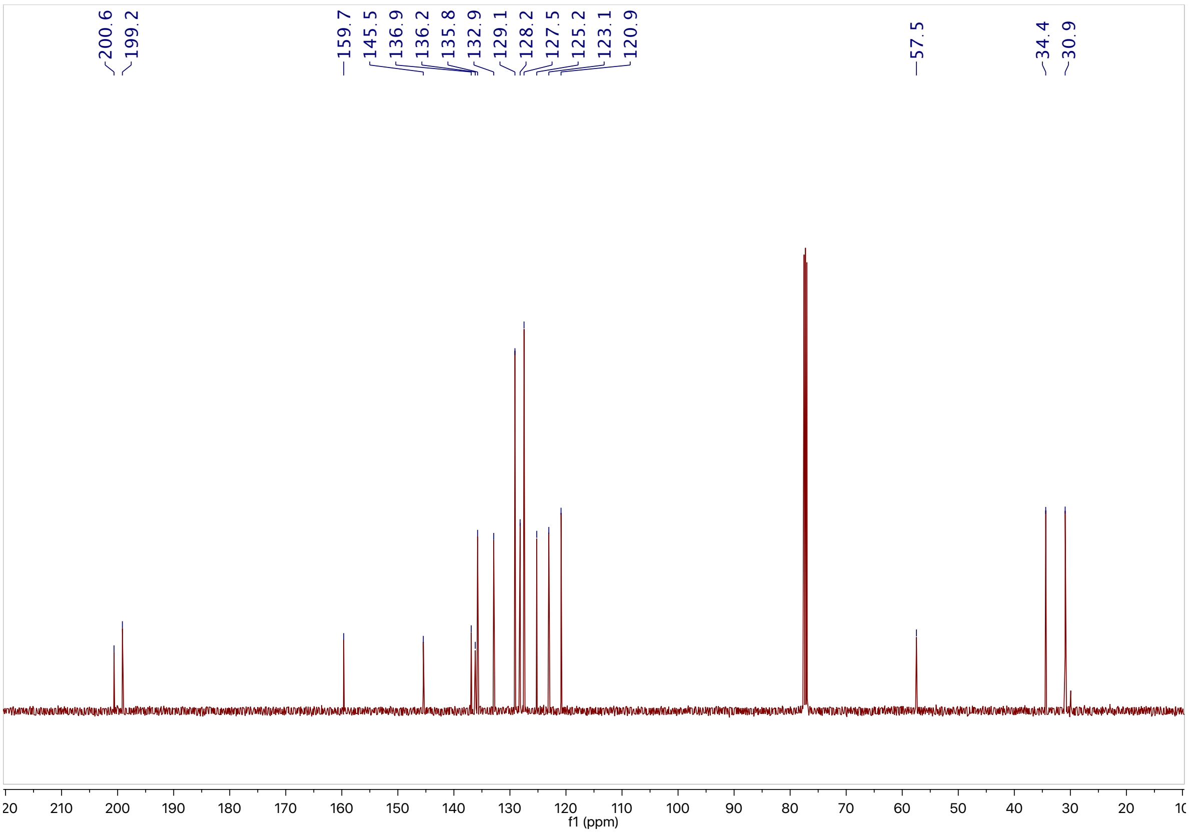
200.6
199.2
159.7
145.5
136.9
136.2
135.8
132.9
129.1
128.2
127.5
125.2
123.1
120.9
57.5
34.4
30.9
f1 (ppm)

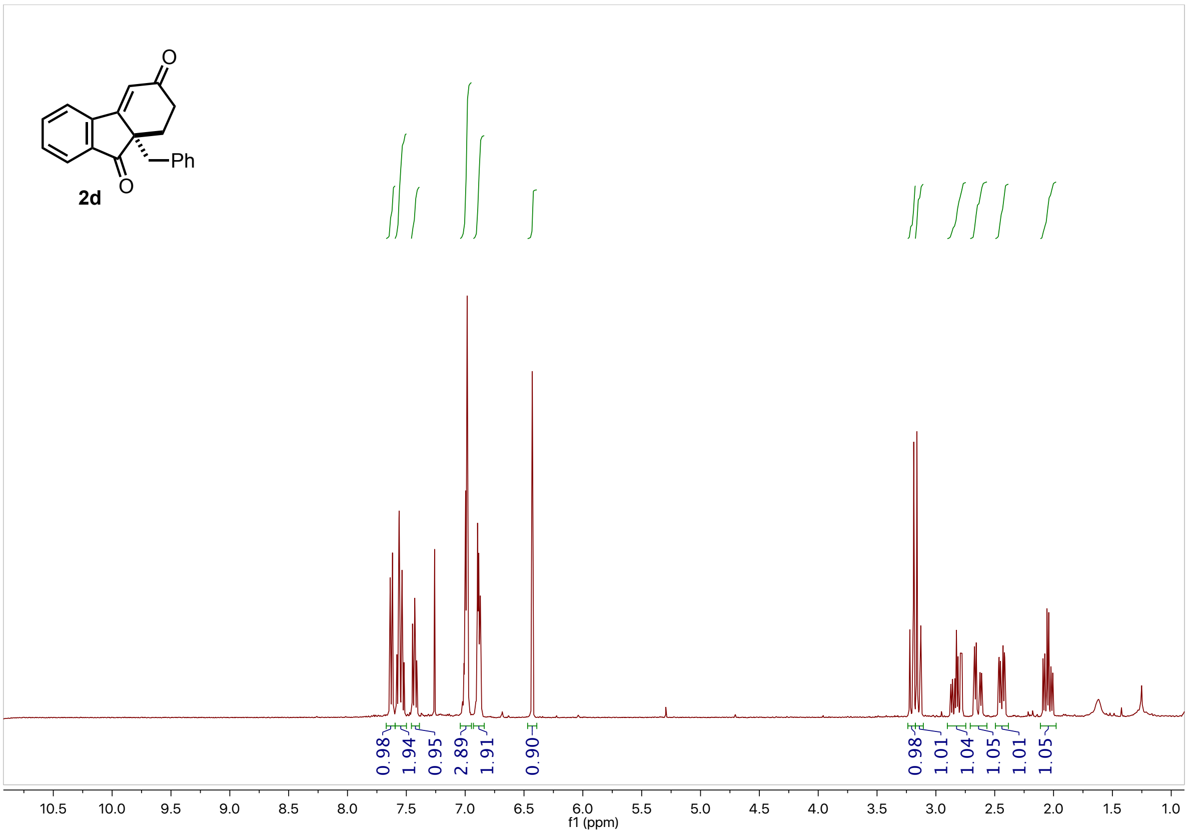
O
O
Ph
2d
0.98
1.94
0.95
2.89
1.91
0.90
0.98
1.01
1.04
1.05
1.01
1.05
10.5 10.0 9.5 9.0 8.5 8.0 7.5 7.0 6.5 6.0 5.5 5.0 4.5 4.0 3.5 3.0 2.5 2.0 1.5 1.0
f1 (ppm)

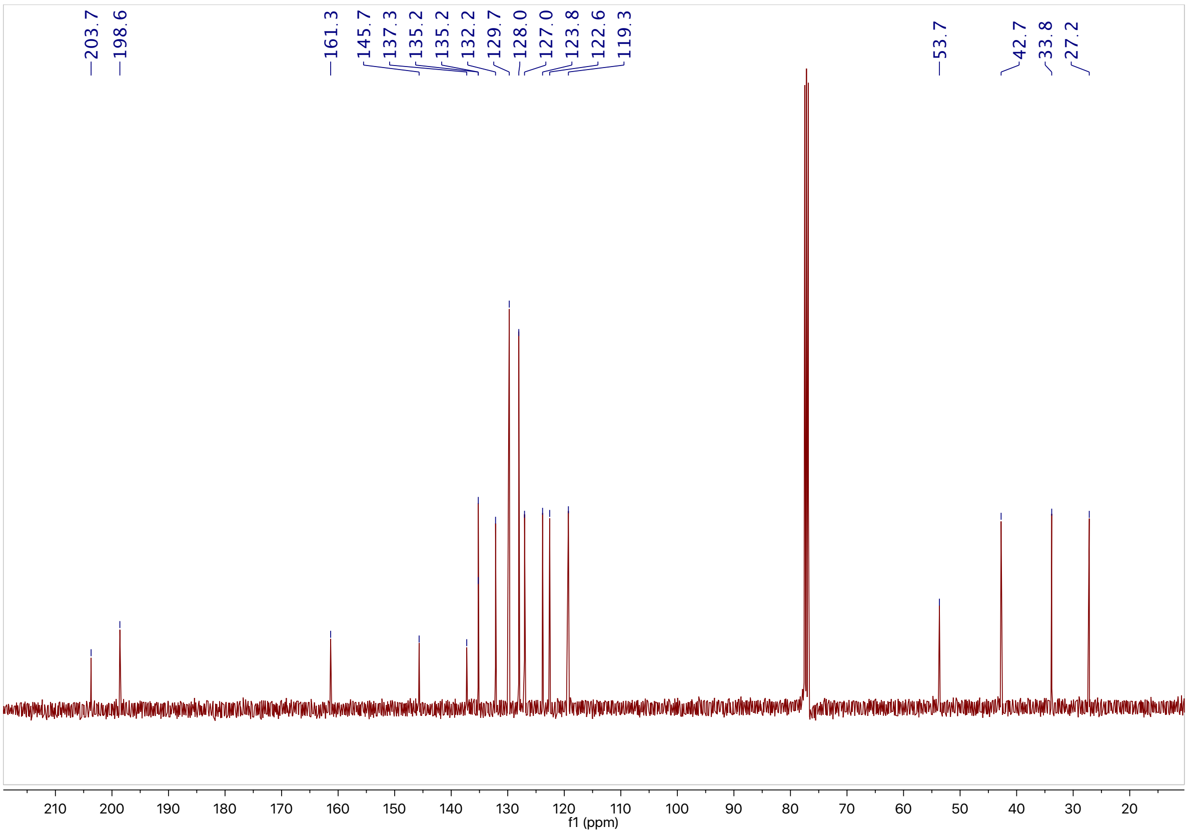
203.7
198.6
161.3
145.7
137.3
135.2
135.2
132.2
129.7
128.0
127.0
123.8
122.6
119.3
53.7
42.7
33.8
27.2
210 200 190 180 170 160 150 140 130 120 110 100 90 80 70 60 50 40 30 20
f1 (ppm)

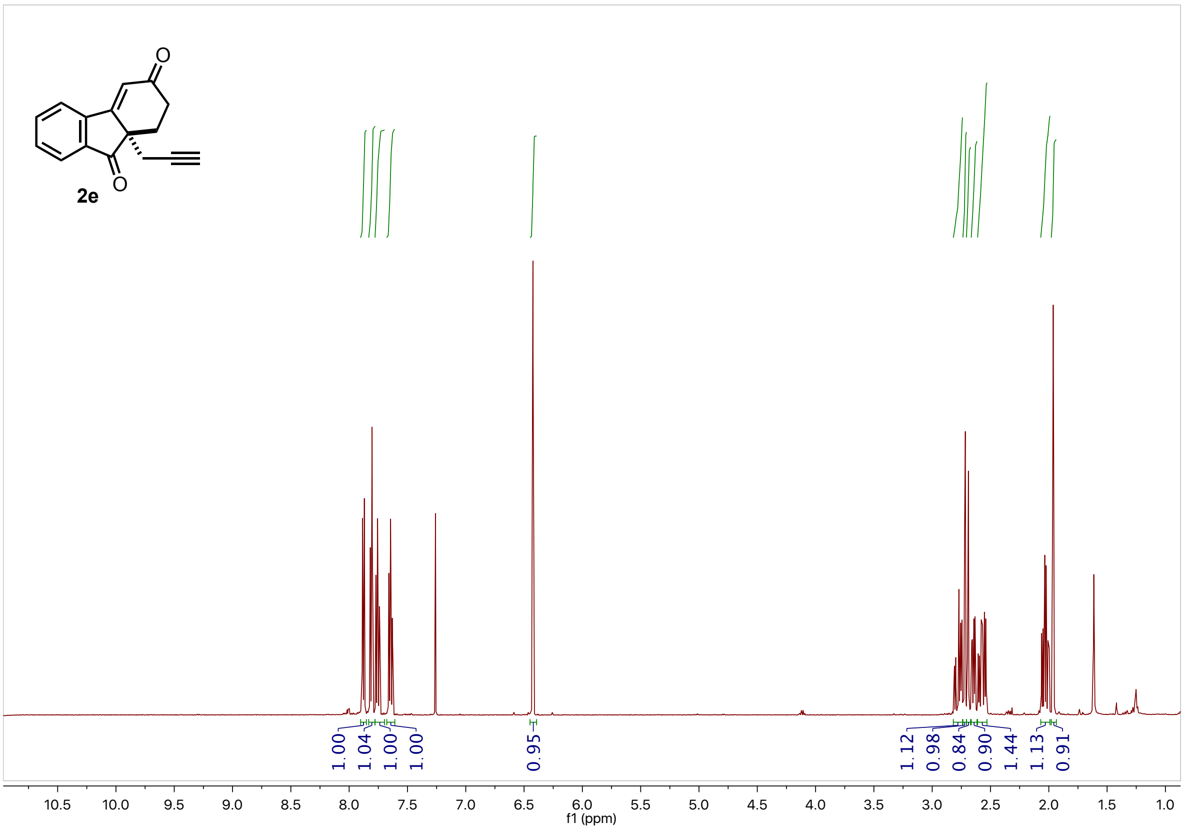
2e
1.00
1.04
1.00
1.00
0.95
1.12
0.98
0.84
0.90
1.44
1.13
0.91
10.5 10.0 9.5 9.0 8.5 8.0 7.5 7.0 6.5 6.0 5.5 5.0 4.5 4.0 3.5 3.0 2.5 2.0 1.5 1.0
f1 (ppm)

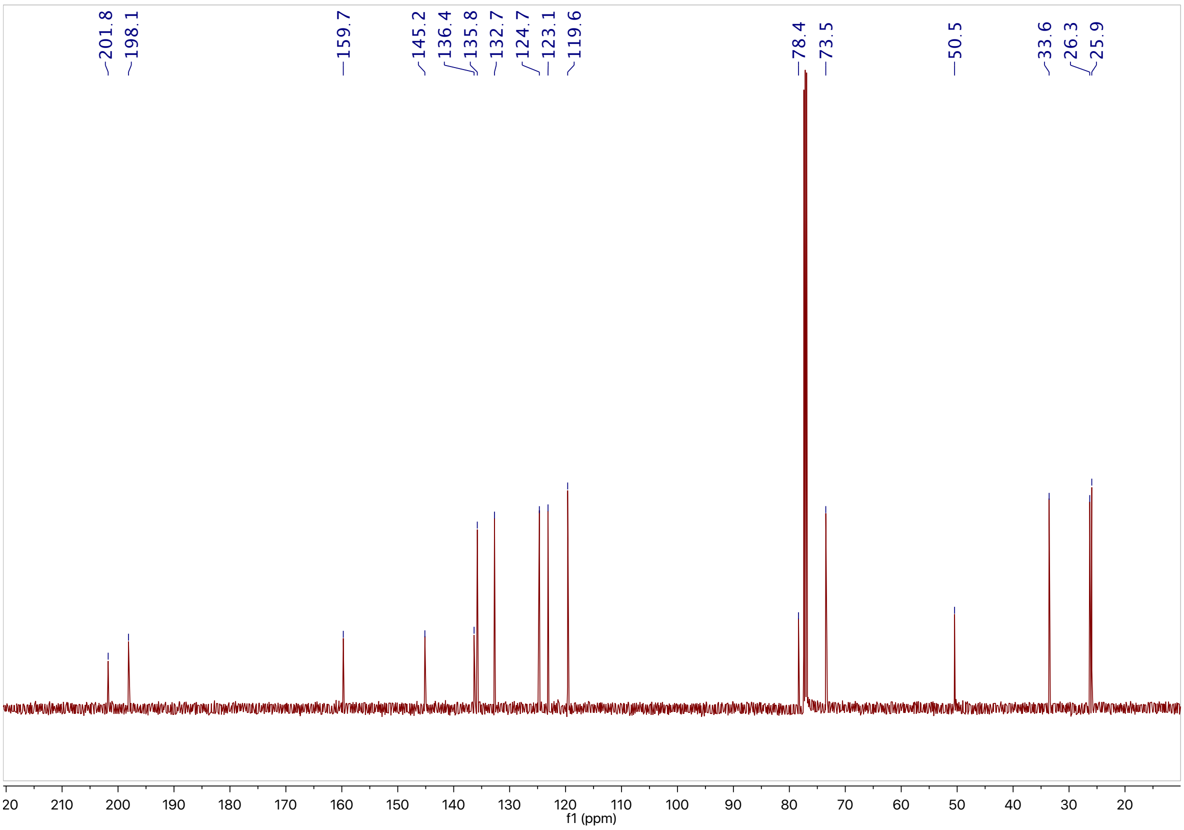
201.8
198.1
159.7
145.2
136.4
135.8
132.7
124.7
123.1
119.6
78.4
73.5
50.5
33.6
26.3
25.9
20 210 200 190 180 170 160 150 140 130 120 110 100 90 80 70 60 50 40 30 20
f1 (ppm)

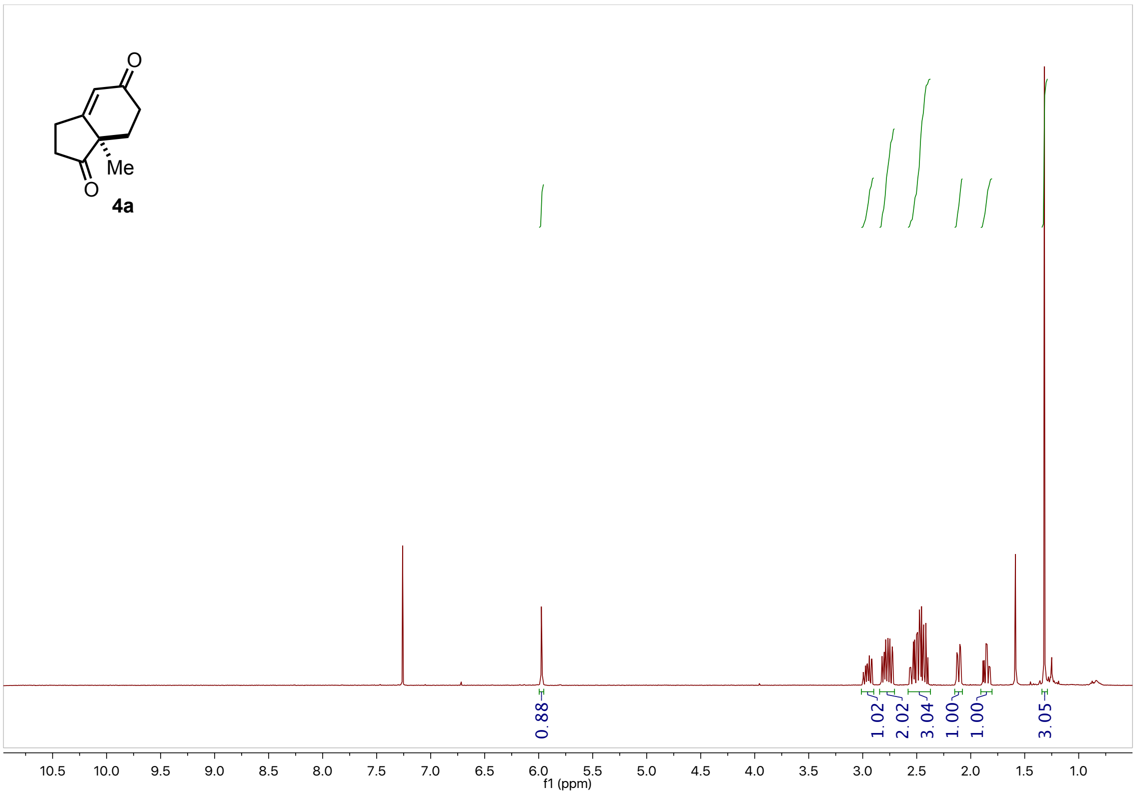
O
Me
O
4a
0.88
1.02
2.02
3.04
1.00
1.00
3.05
10.5 10.0 9.5 9.0 8.5 8.0 7.5 7.0 6.5 6.0 5.5 5.0 4.5 4.0 3.5 3.0 2.5 2.0 1.5 1.0
f1 (ppm)

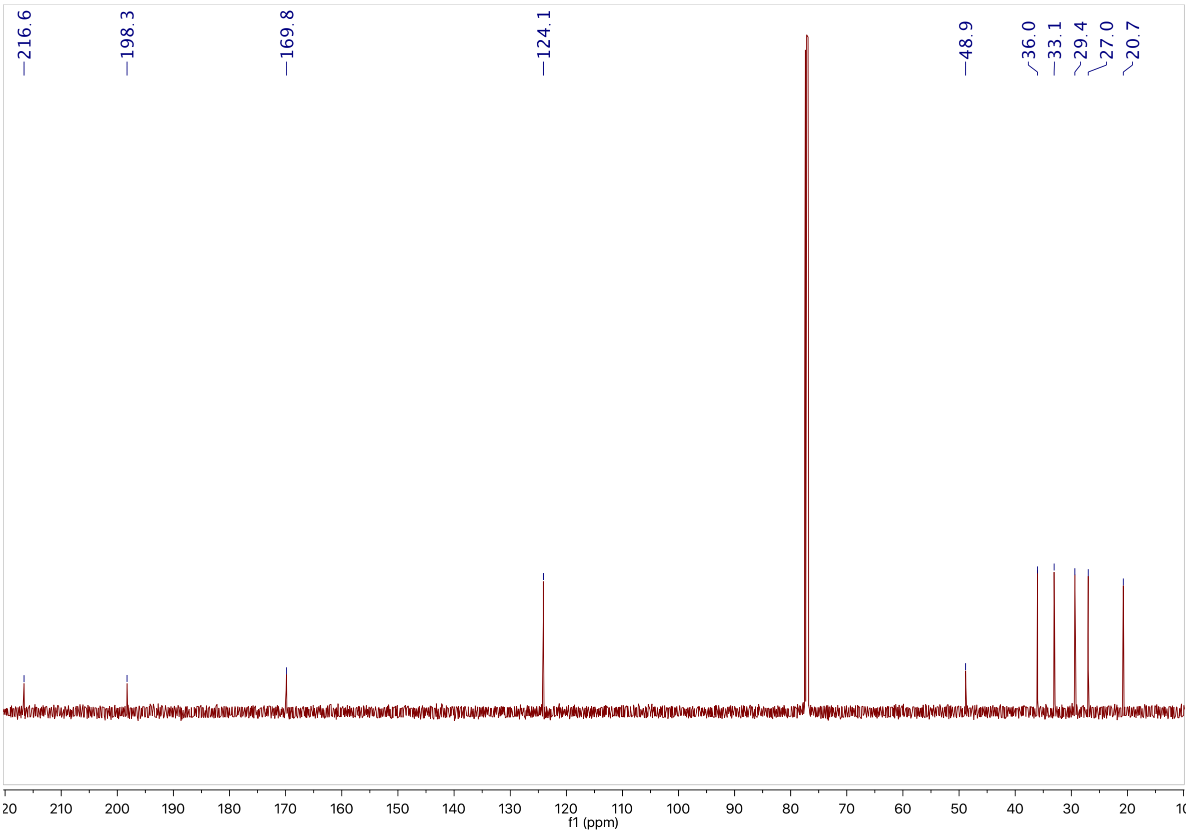
216.6
198.3
169.8
124.1
48.9
36.0
33.1
29.4
27.0
20.7
210 200 190 180 170 160 150 140 130 120 110 100 90 80 70 60 50 40 30 20
f1 (ppm)

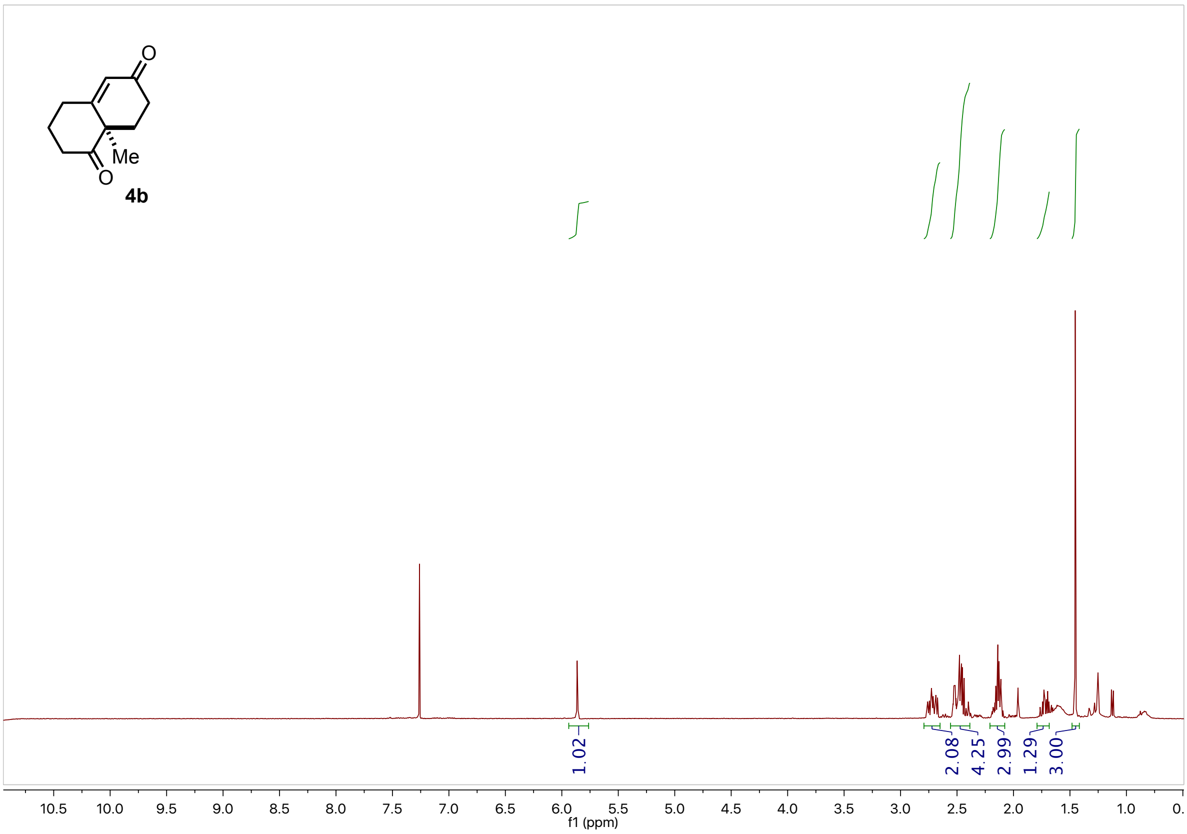

Me
4b
1.02
2.08
4.25
2.99
1.29
3.00
10.5 10.0 9.5 9.0 8.5 8.0 7.5 7.0 6.5 6.0 5.5 5.0 4.5 4.0 3.5 3.0 2.5 2.0 1.5 1.0 0.
f1 (ppm)

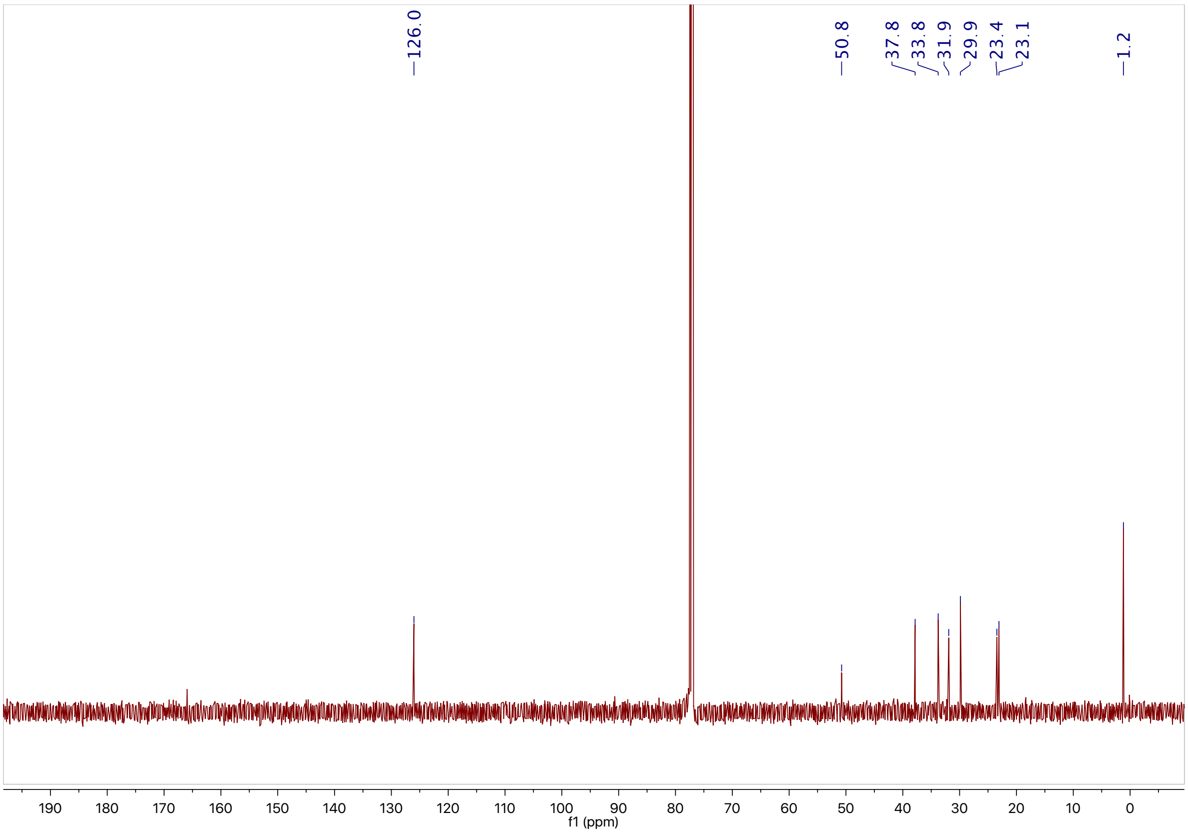

126.0
50.8
37.8
33.8
31.9
29.9
23.4
23.1
1.2
190 180 170 160 150 140 130 120 110 100 90 80 70 60 50 40 30 20 10 0
f1 (ppm)

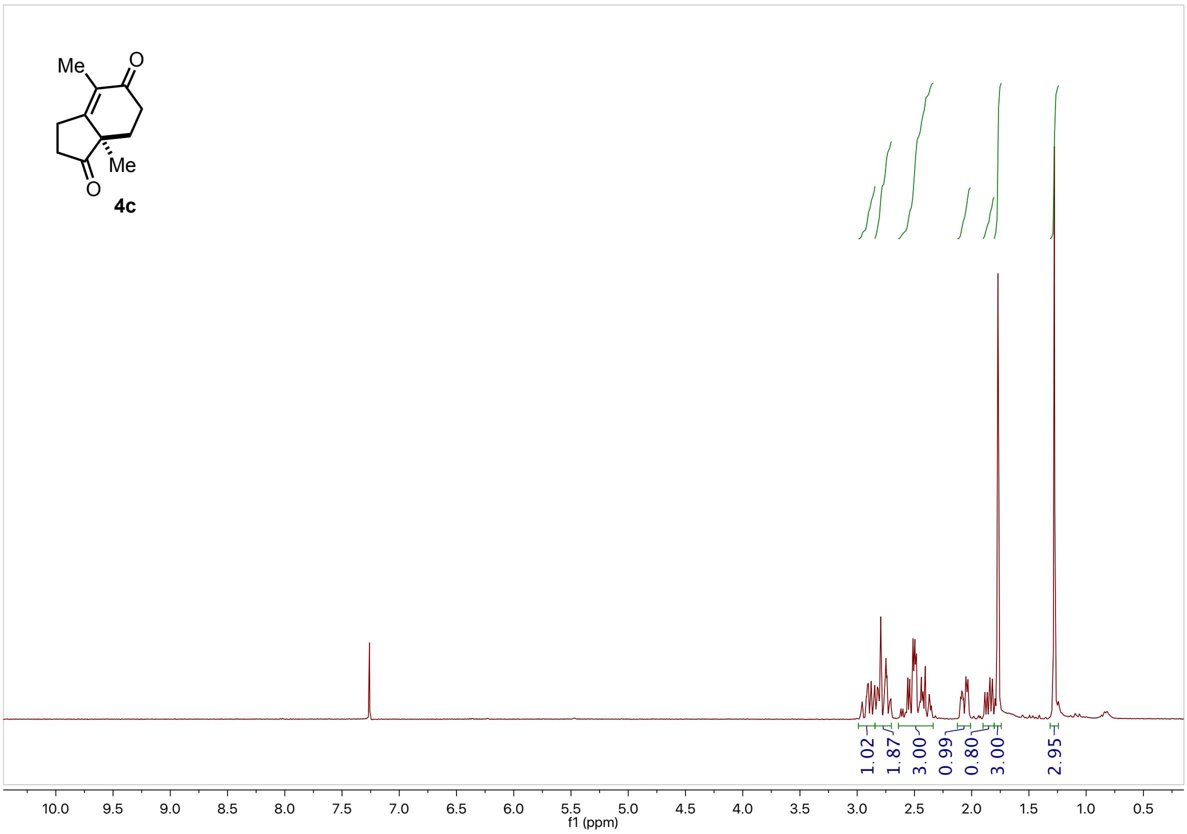

Me
O
Me
O
4c
1.02
1.87
3.00
0.99
0.80
3.00
2.95
10.0 9.5 9.0 8.5 8.0 7.5 7.0 6.5 6.0 5.5 5.0 4.5 4.0 3.5 3.0 2.5 2.0 1.5 1.0 0.5
f1 (ppm)

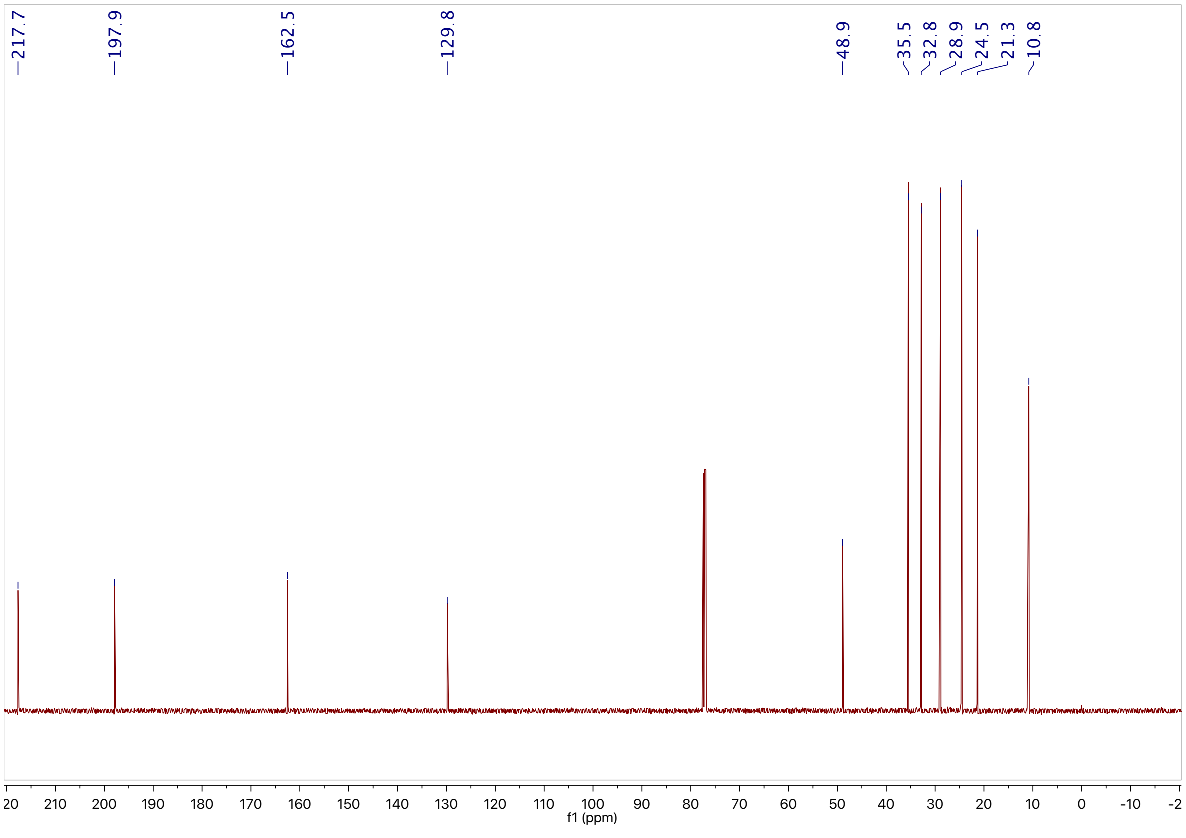

217.7
197.9
162.5
129.8
48.9
35.5
32.8
28.9
24.5
21.3
10.8
20 210 200 190 180 170 160 150 140 130 120 110 100 90 80 70 60 50 40 30 20 10 0 -10 -2
f1 (ppm)

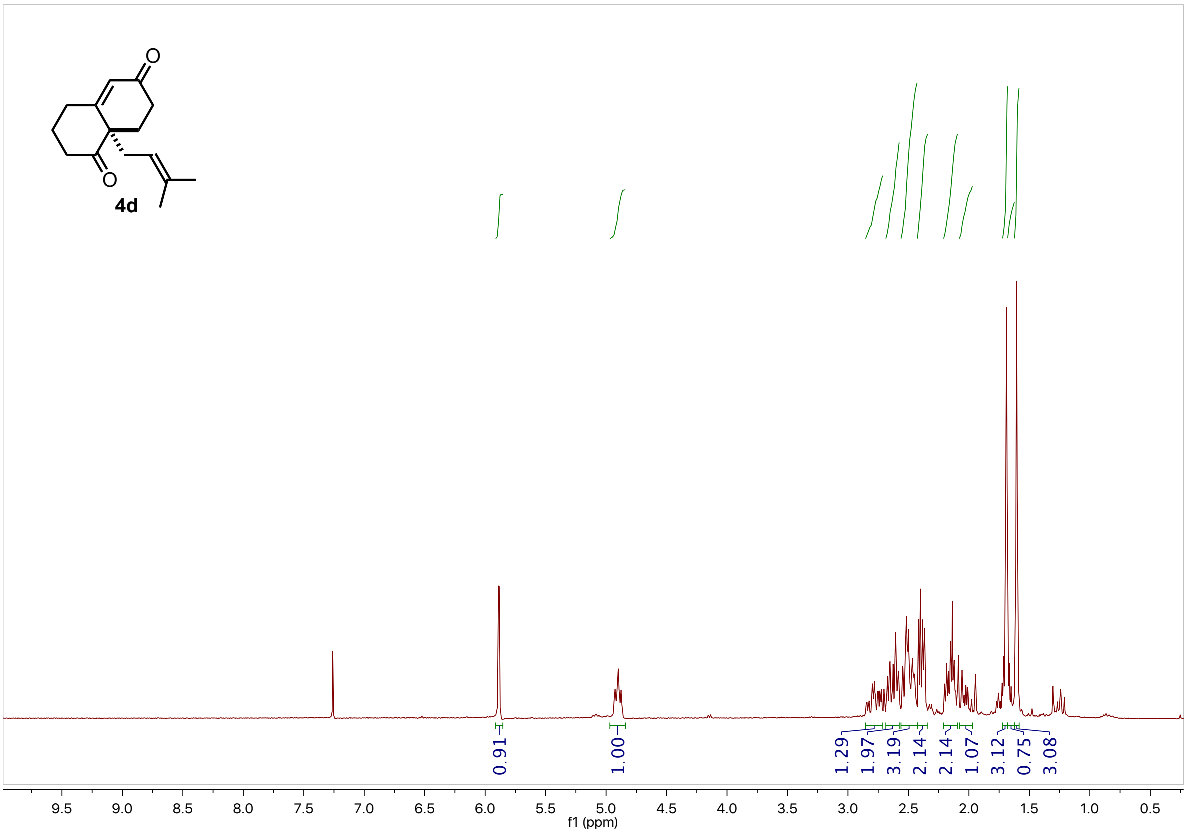
4d
0.91
1.00
1.29
1.97
3.19
2.14
2.14
1.07
3.12
0.75
3.08
9.5 9.0 8.5 8.0 7.5 7.0 6.5 6.0 5.5 5.0 4.5 4.0 3.5 3.0 2.5 2.0 1.5 1.0 0.5
f1 (ppm)

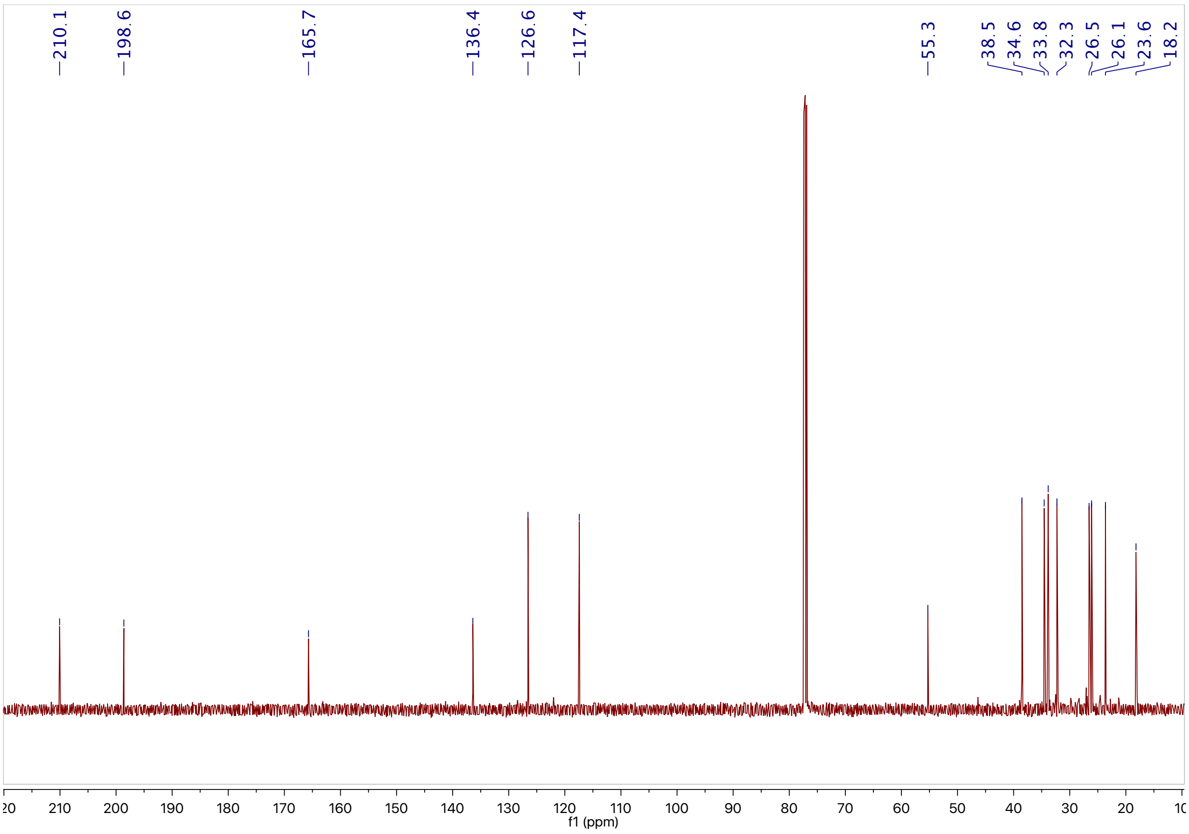
210.1
198.6
165.7
136.4
126.6
117.4
55.3
38.5
34.6
33.8
32.3
26.5
26.1
23.6
18.2
210 200 190 180 170 160 150 140 130 120 110 100 90 80 70 60 50 40 30 20
f1 (ppm)

6. HPLC Chromatograms

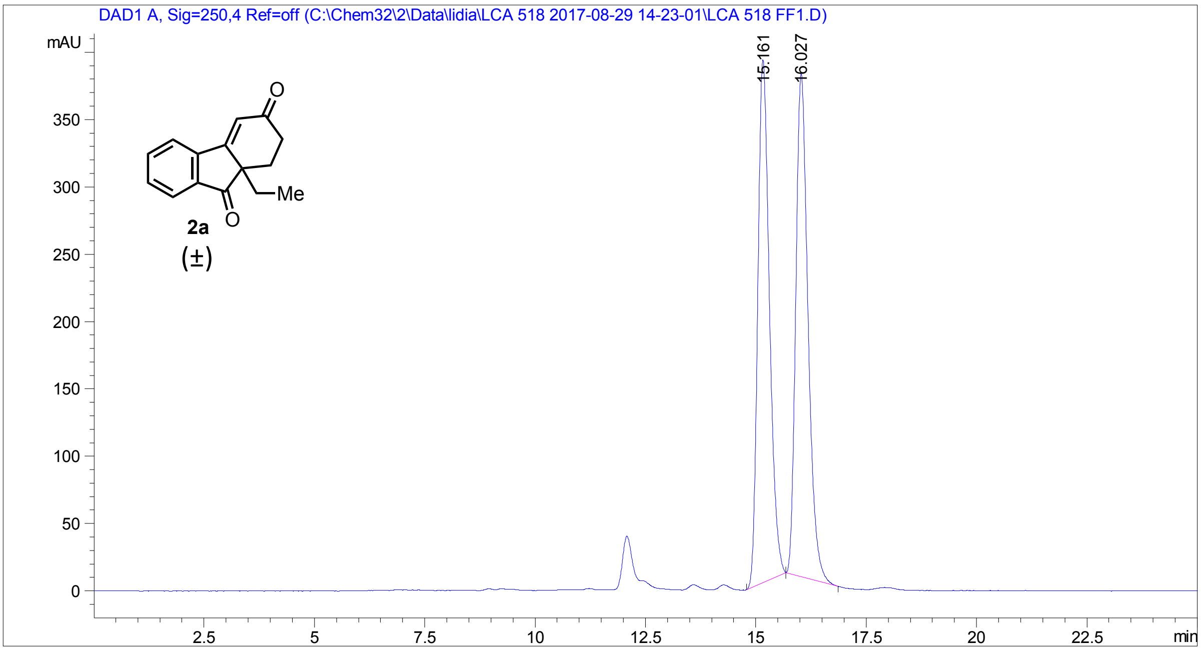

Peak #	Ret Time [min]	Type	Width [min]	Area [mAU*s]	Height [mAU]	Area %
1	15.161	BB	0.2818	7112.54199	388.14499	49.8096
2	16.027	BB	0.2960	7166.90527	373.32489	50.1904

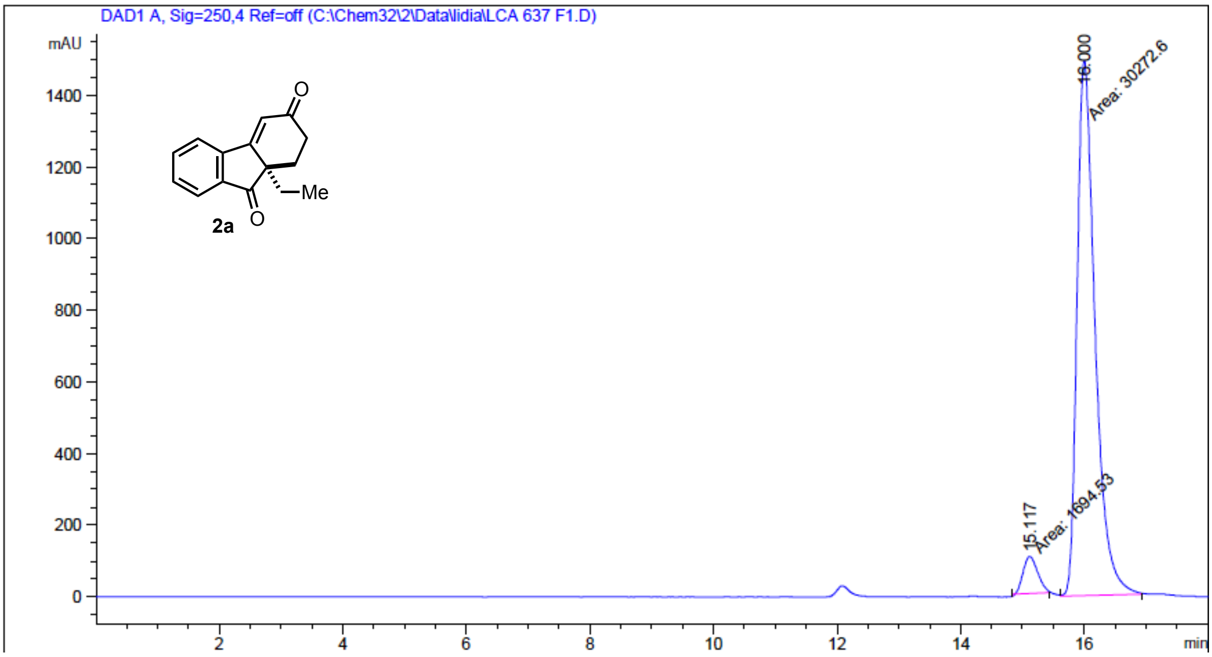

Peak #	RetTime [min]	Type	Width [min]	Area [mAU*s]	Height [mAU]	Area %
1	15.117	MM	0.2739	1694.52747	103.12574	5.3008
2	16.000	MM	0.3378	3.02726e4	1493.61218	94.6992

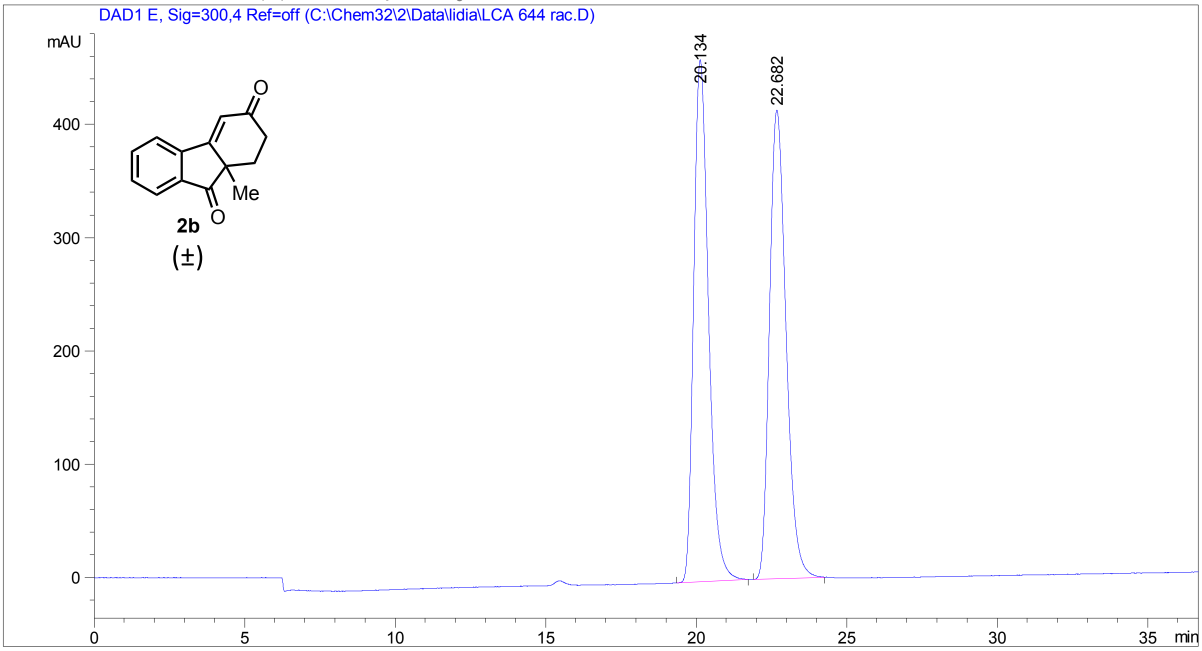

Peak #	Ret Time [min]	Type	Width [min]	Area [mAU*s]	Height [mAU]	Area %
1	20.134	BB	0.5327	1.58197e4	460.61551	50.0556
2	22.682	BB	0.5912	1.57846e4	413.63757	49.9444

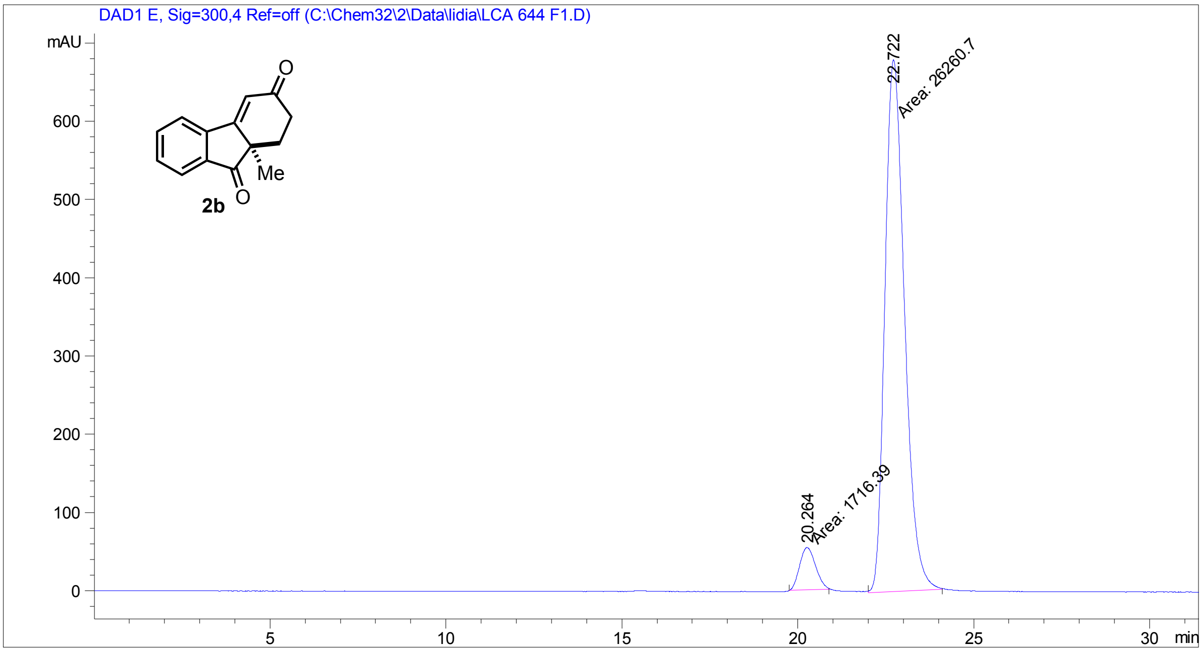

Peak #	Ret Time [min]	Type	Width [min]	Area [mAU*s]	Height [mAU]	Area %
1	20.264	MM	0.5270	1716.38916	54.28528	6.1350
2	22.722	MM	0.6441	2.62607e4	679.49506	93.8650

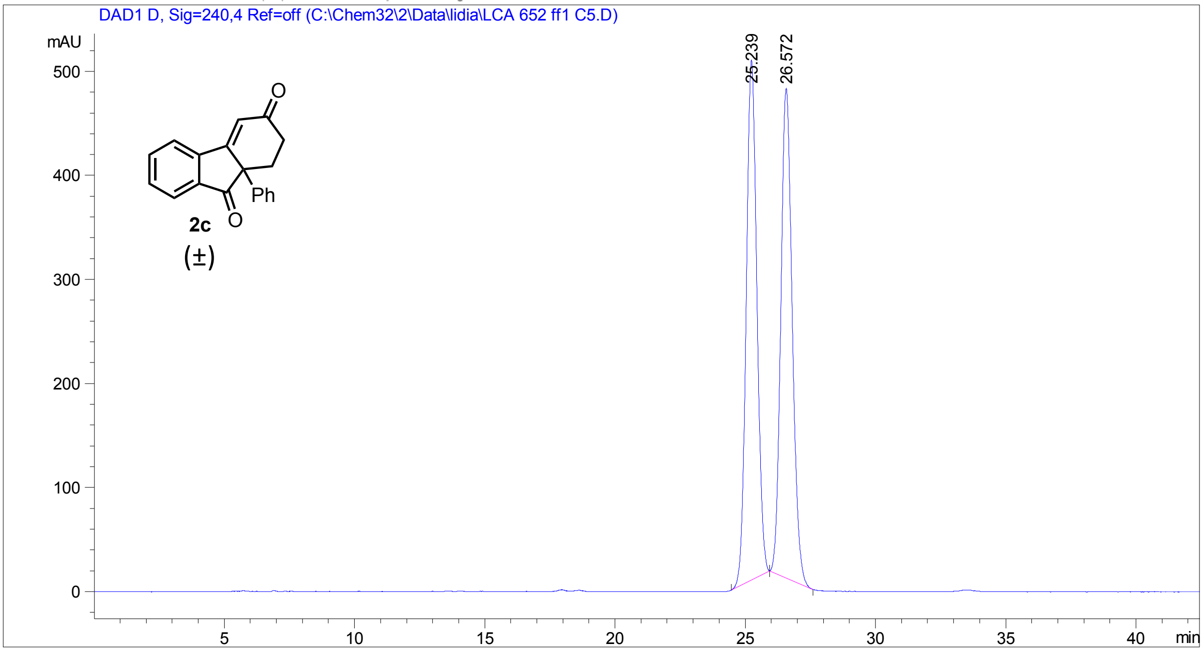

Peak #	Ret Time [min]	Type	Width [min]	Area [mAU*s]	Height [mAU]	Area %
1	25.239	BB	0.4475	1.42008e4	499.79587	49.8195
2	26.572	BB	0.4726	1.43037e4	470.60620	50.1805

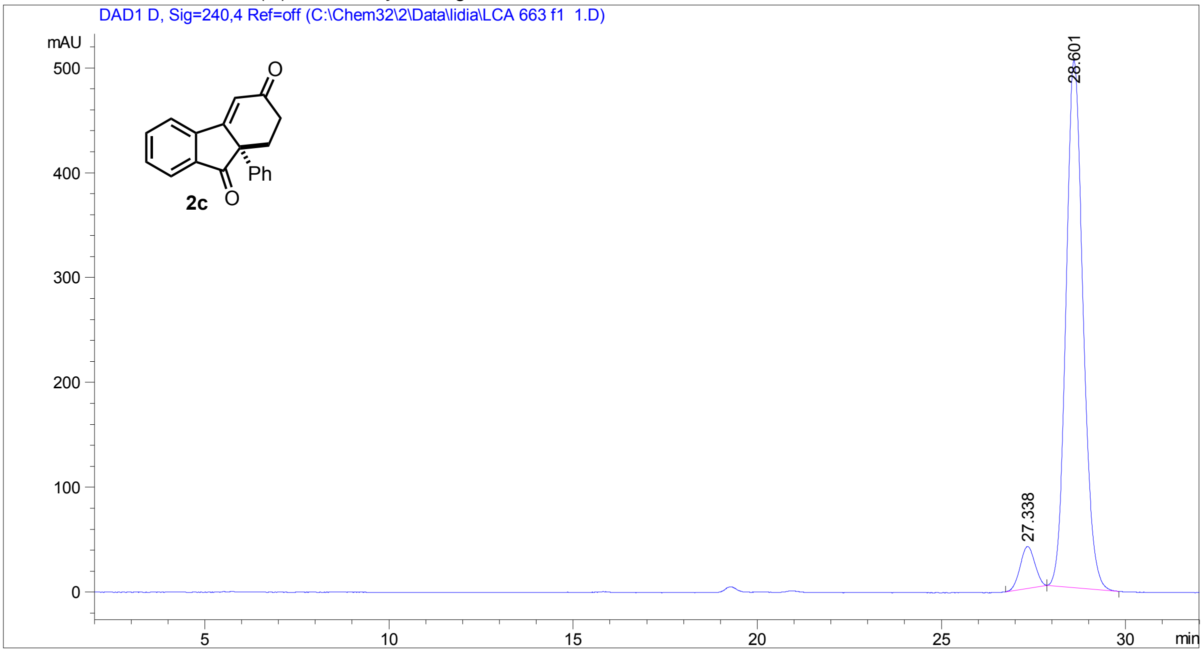

Peak #	Ret Time [min]	Type	Width [min]	Area [mAU*s]	Height [mAU]	Area %
1	27.338	BB	0.4087	1103.87598	39.91023	6.3822
2	28.601	BB	0.5035	1.61924e4	503.04770	93.6178

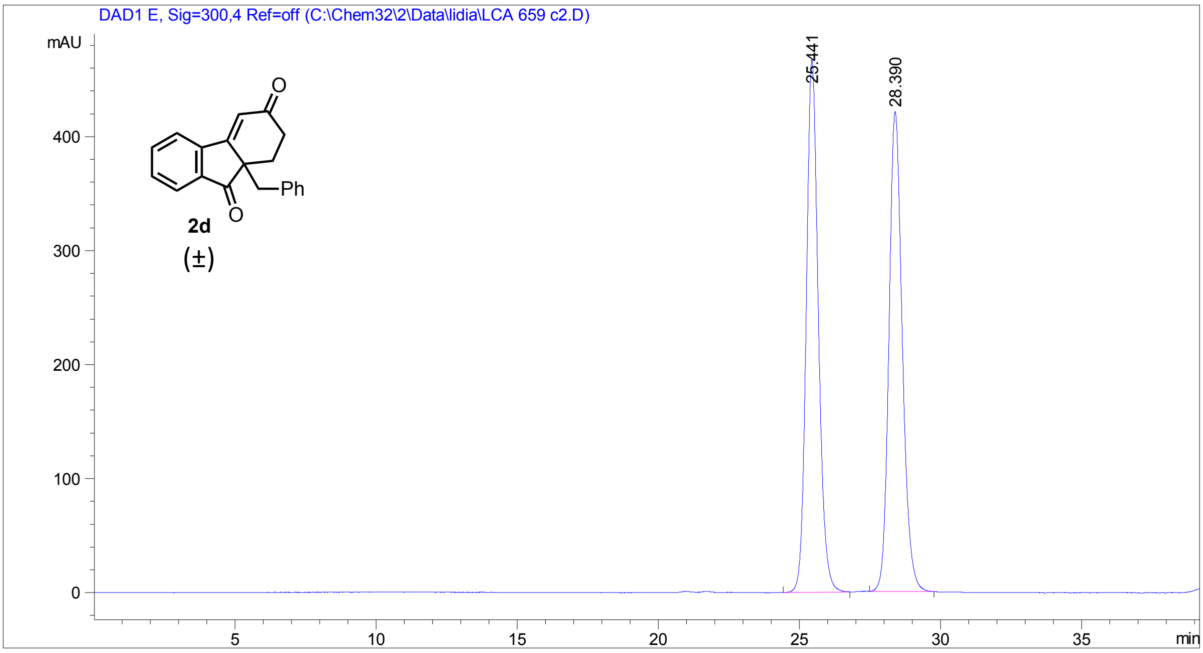

Peak #	Ret Time [min]	Type	Width [min]	Area [mAU*s]	Height [mAU]	Area %
1	25.441	BB	0.4513	1.38929e4	466.70422	50.1218
2	28.390	BB	0.5029	1.38253e4	421.21667	49.8782

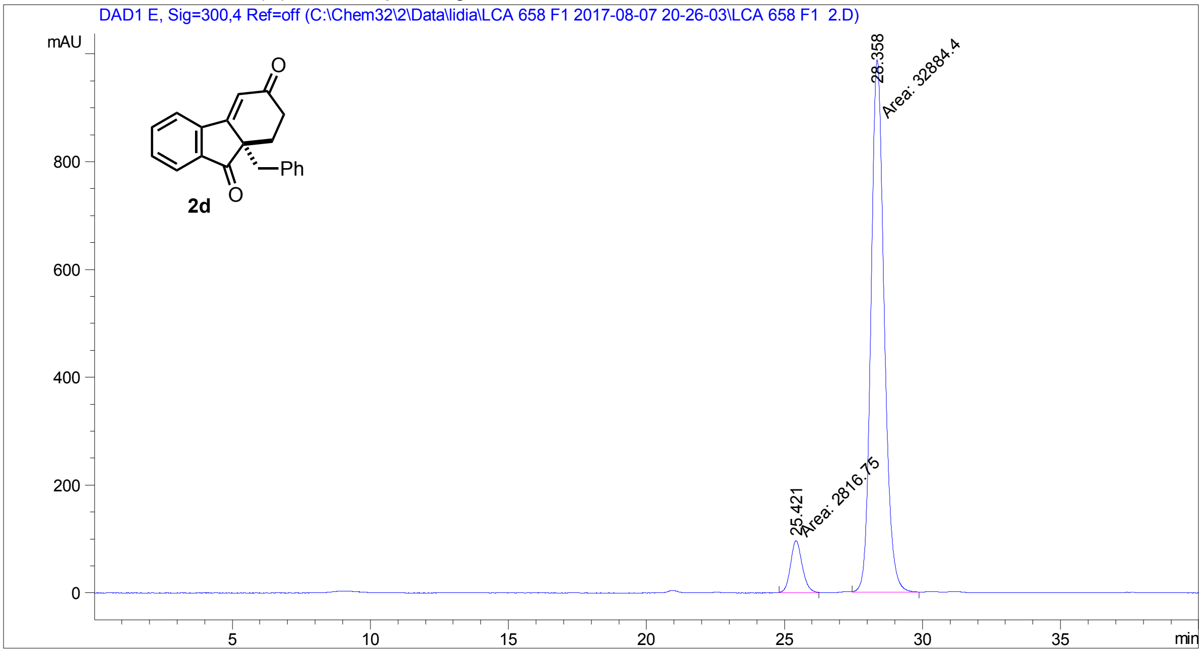

Peak #	Ret Time [min]	Type	Width [min]	Area [mAU*s]	Height [mAU]	Area %
1	25.421	MM	0.4892	2816.75000	95.96868	7.8898
2	28.358	MM	0.5551	3.28844e4	987.41382	92.1102

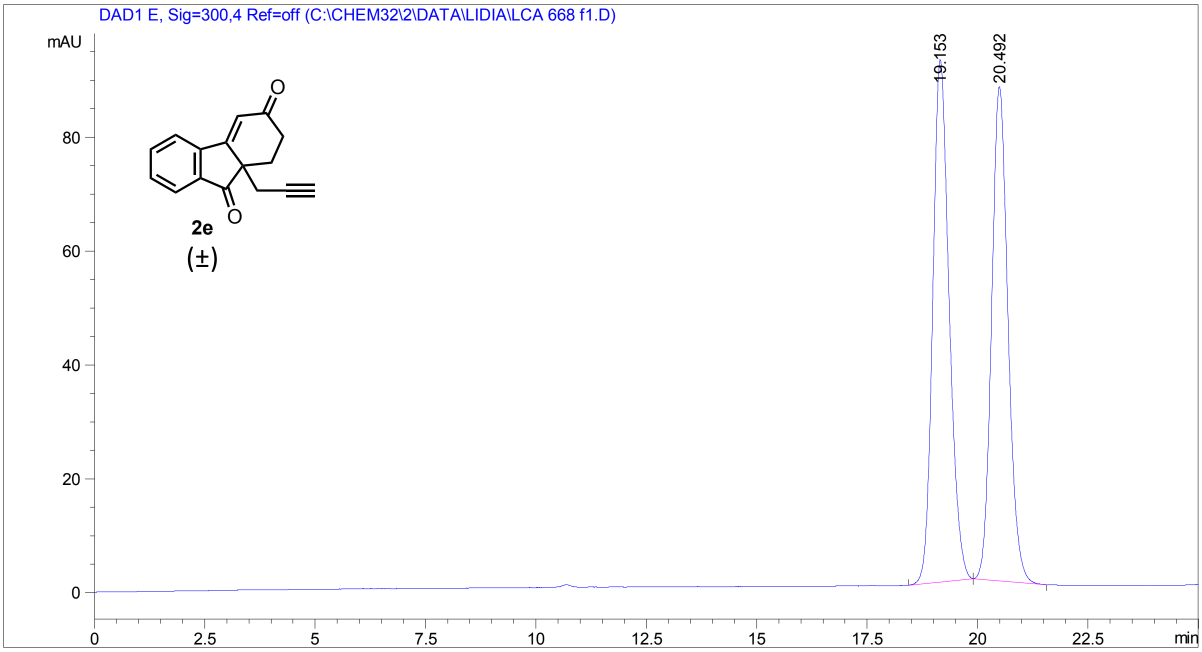

Peak #	Ret Time [min]	Type	Width [min]	Area [mAU*s]	Height [mAU]	Area %
1	19.153	BB	0.3948	2365.06958	91.71776	51.4605
2	20.492	BB	0.3940	2230.82324	86.75027	48.5395

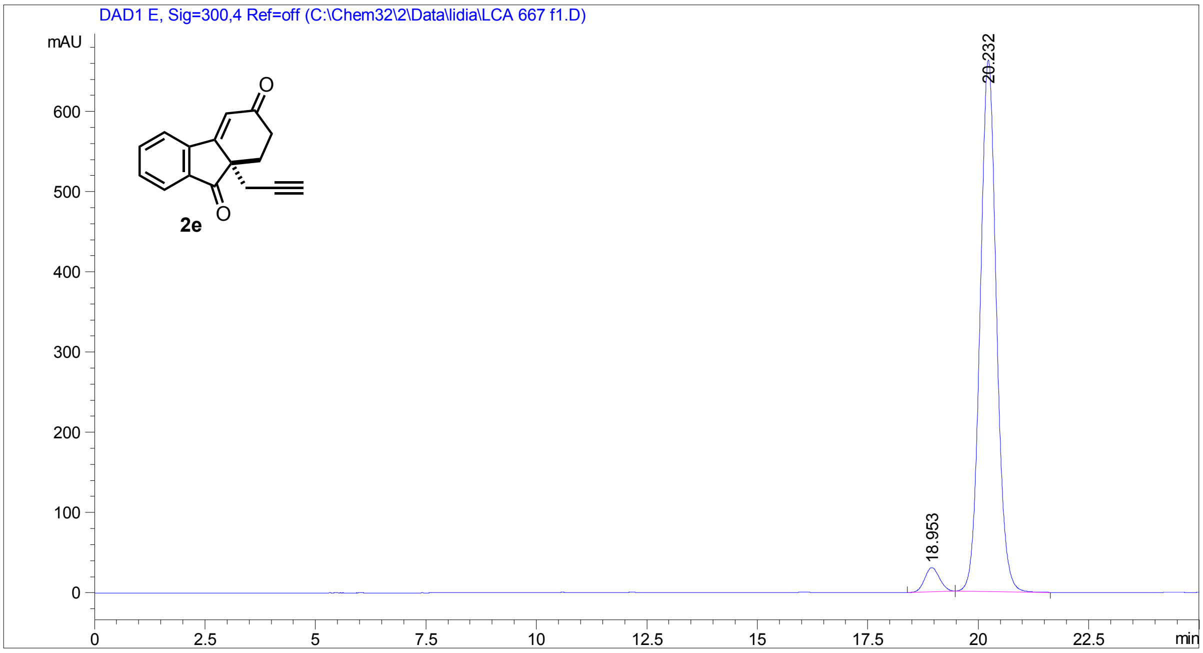

Peak #	Ret Time [min]	Type	Width [min]	Area [mAU*s]	Height [mAU]	Area %
1	18.953	BB	0.3649	706.74945	30.21742	3.9985
2	20.232	BB	0.3947	1.69686e4	662.71729	96.0015

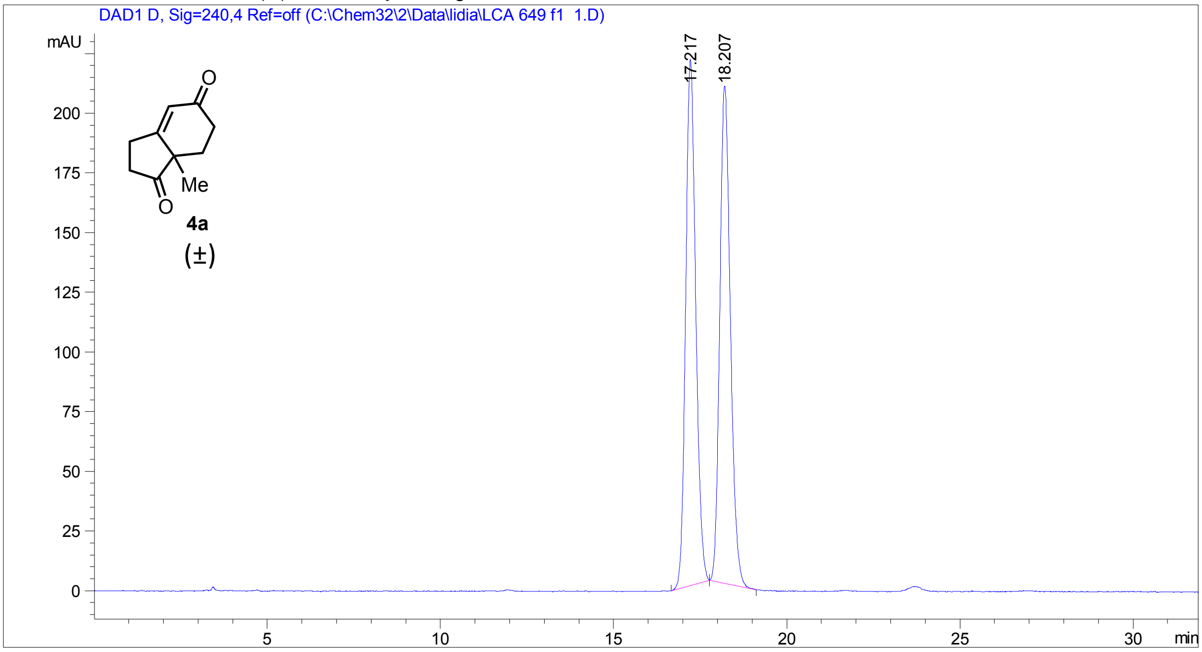

Peak #	Ret Time [min]	Type	Width [min]	Area [mAU*s]	Height [mAU]	Area %
1	17.217	BB	0.3074	4442.87939	220.18291	50.0327
2	18.207	BB	0.3304	4437.07813	208.39751	49.9673

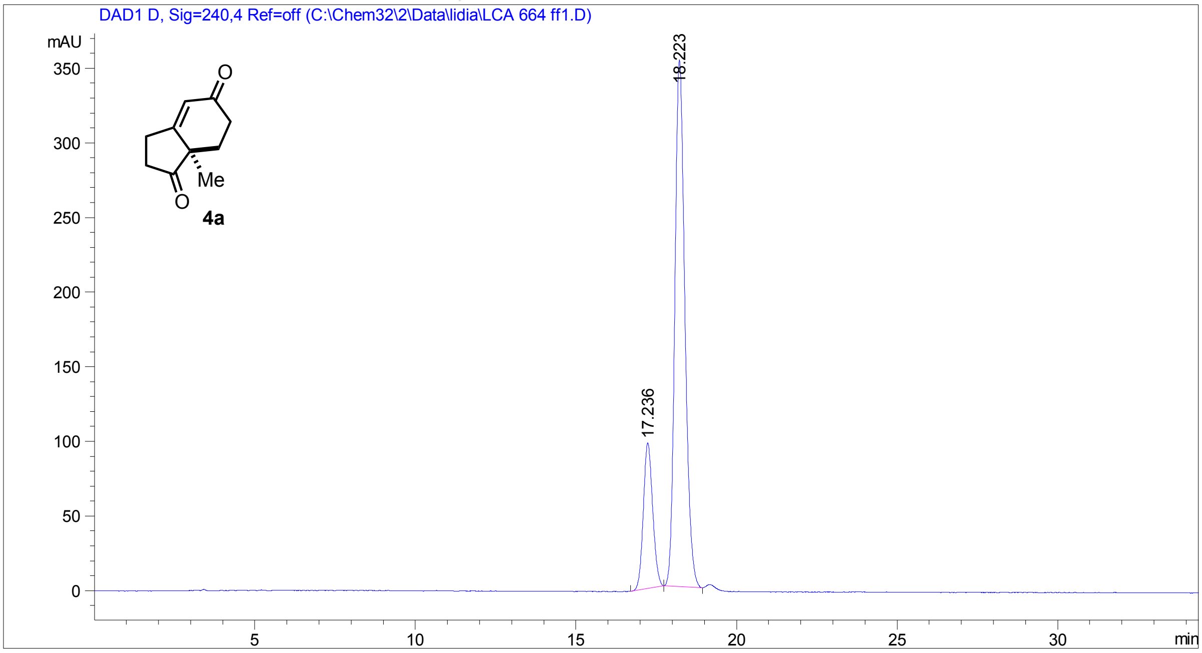

Peak #	Ret Time [min]	Type	Width [min]	Area [mAU*s]	Height [mAU]	Area %
1	17.236	BB	0.3059	1973.06824	97.57182	20.4902
2	18.223	BB	0.3331	7656.27148	352.92709	79.5098

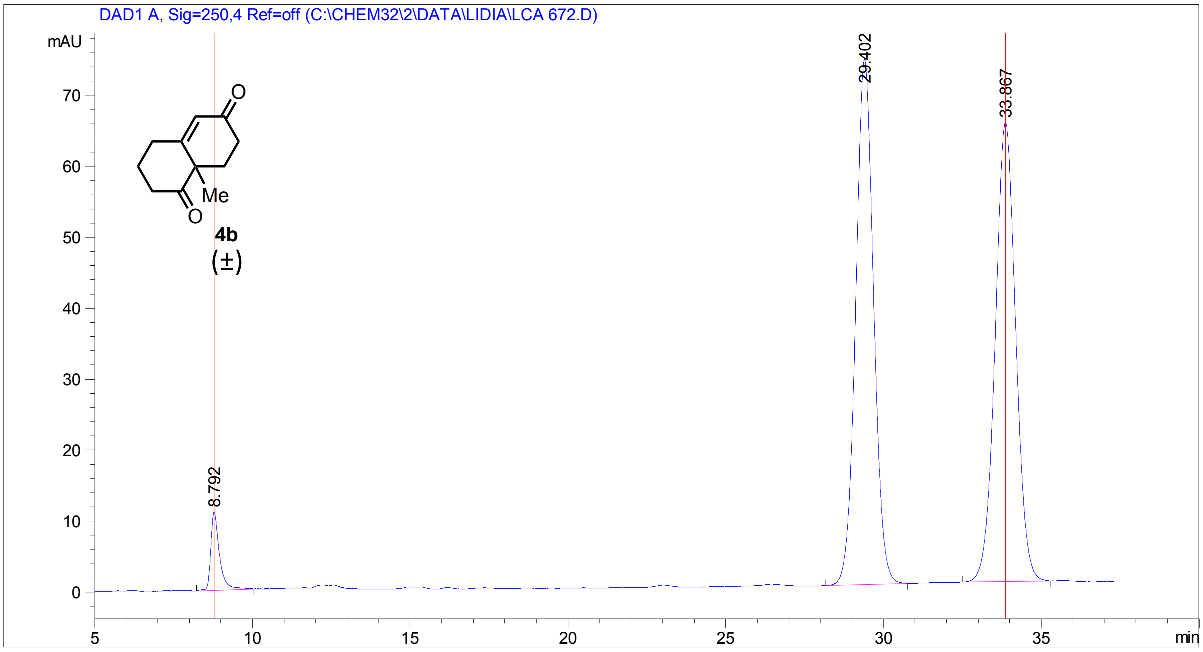

Peak #	Ret Time [min]	Type	Width [min]	Area [mAU*s]	Height [mAU]	Area %
1	8.792	BB	0.2838	211.11017	11.00668	3.4913
2	29.402	BB	0.6061	2917.20947	73.97626	48.2444
3	33.867	BB	0.6964	2918.41724	64.67725	48.2643

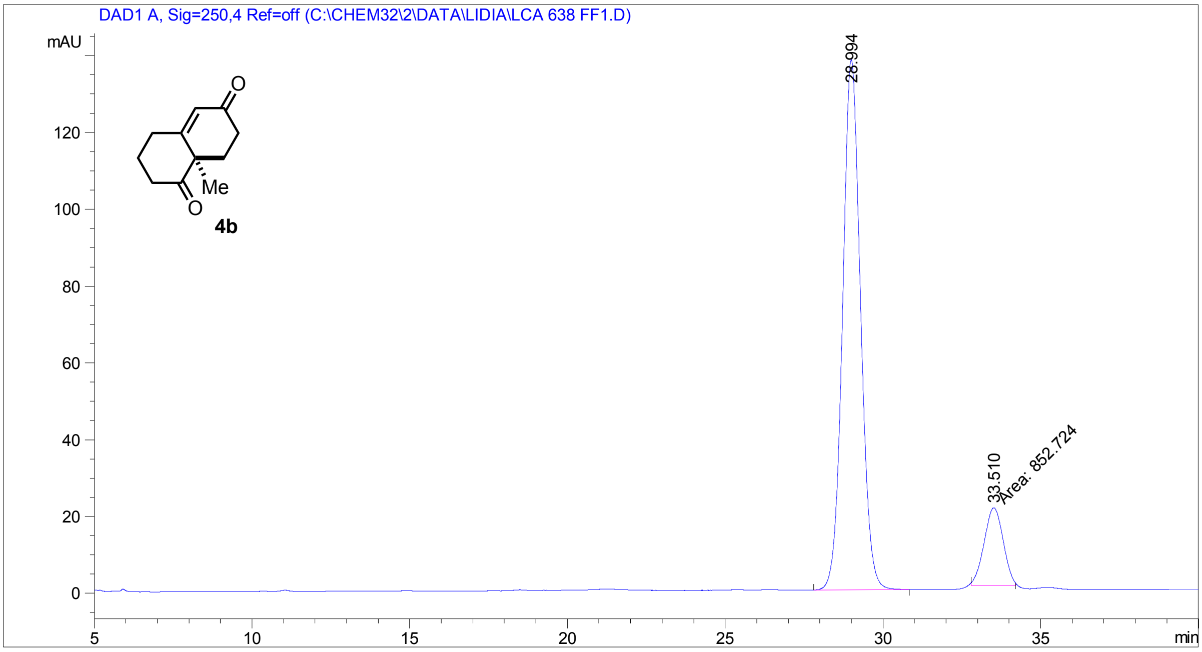

Peak #	Ret Time [min]	Type	Width [min]	Area [mAU*s]	Height [mAU]	Area %
1	28.994	BB	0.6037	5438.40967	138.01724	86.4456
2	33.510	MM	0.6994	852.72363	20.31983	13.5544

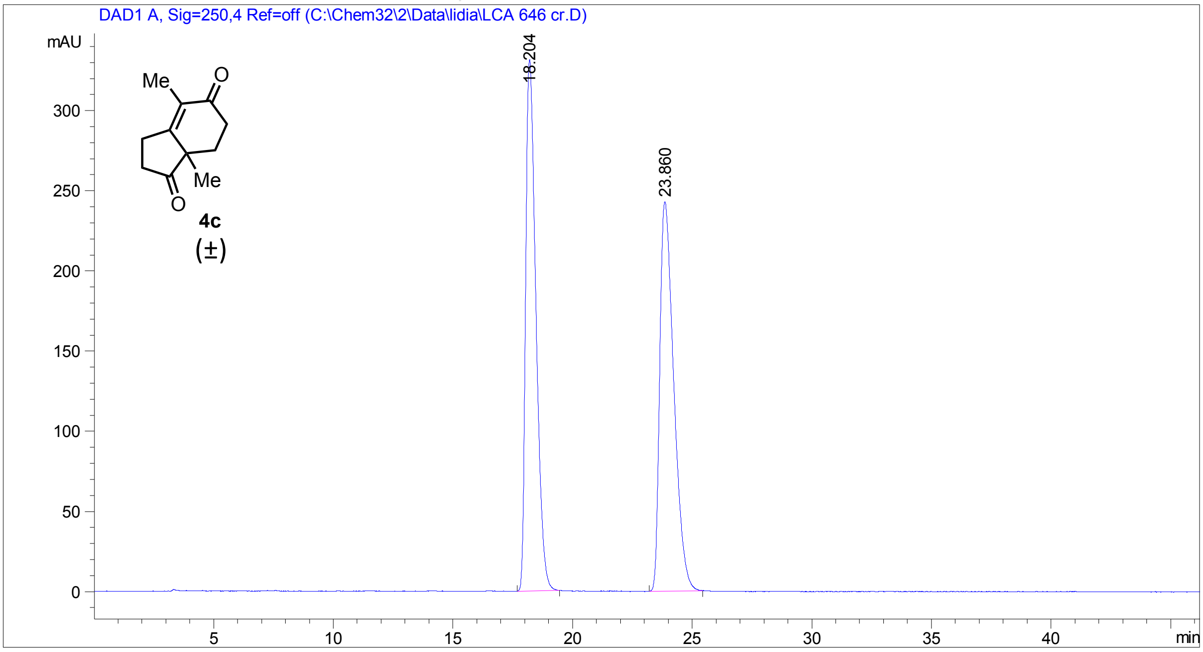

Peak #	Ret Time [min]	Type	Width [min]	Area [mAU*s]	Height [mAU]	Area %
1	18.204	BB	0.4599	9879.79688	331.32367	49.9872
2	23.860	BB	0.6086	9884.86523	243.00079	50.0128

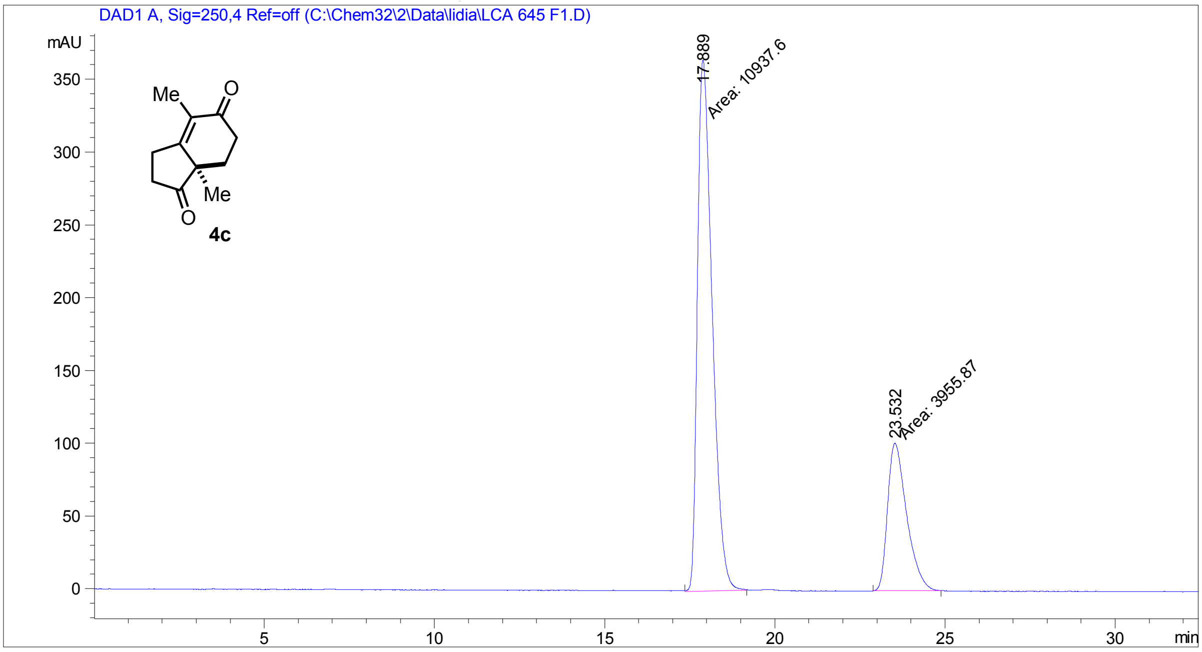

Peak #	Ret Time [min]	Type	Width [min]	Area [mAU*s]	Height [mAU]	Area %
1	17.889	MM	0.4996	1.09376e4	364.88327	73.4389
2	23.532	MM	0.6503	3955.86987	101.38622	26.5611

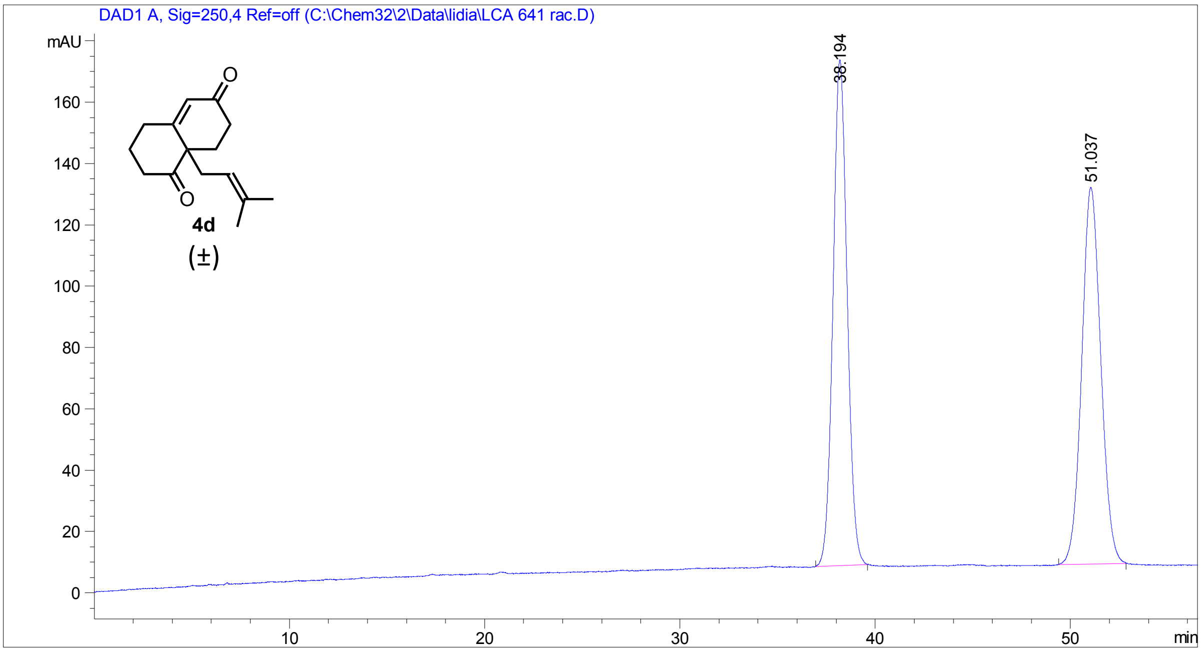

Peak #	Ret Time [min]	Type	Width [min]	Area [mAU*s]	Height [mAU]	Area %
1	38.194	BB	0.7513	8271.92578	164.88728	49.8589
2	51.037	BB	0.8747	8318.74512	122.74912	50.1411

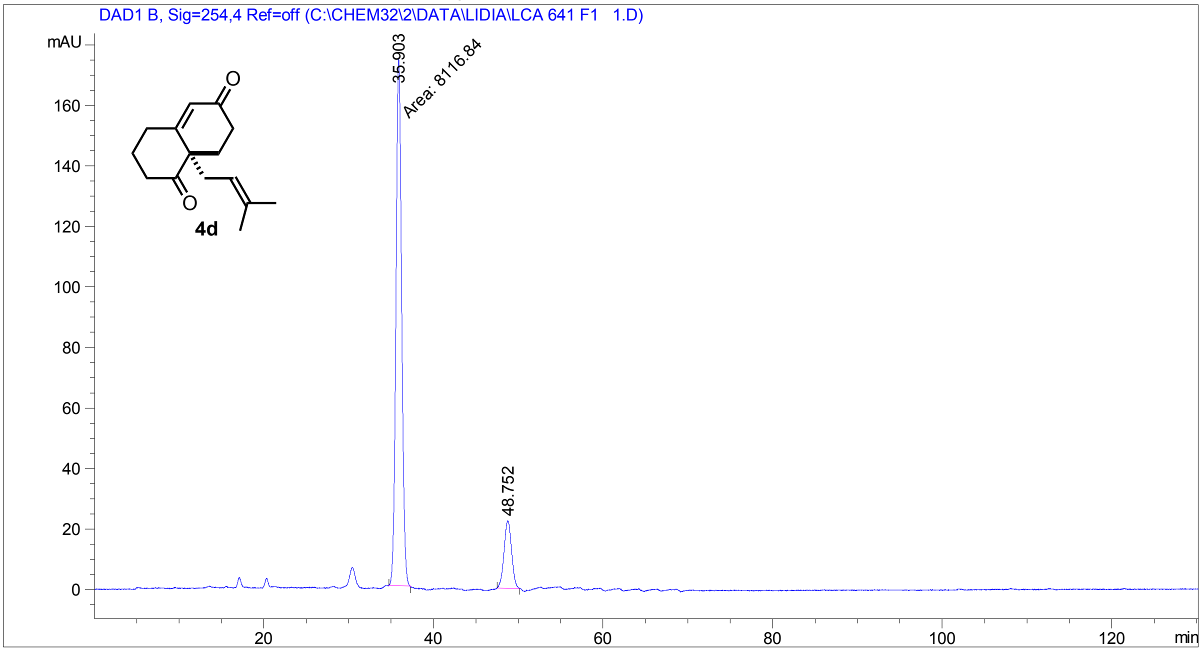

Peak #	Ret Time [min]	Type	Width [min]	Area [mAU*s]	Height [mAU]	Area %
1	35.903	MM	0.7780	8116.84424	173.88992	85.3737
2	48.752	BB	0.7335	1390.58728	22.42321	14.6263
